# Effects of perinatal nutrition supplementation and early weaning on serum biochemistry, metabolomics, and reproduction in yaks

**DOI:** 10.3389/fvets.2024.1443856

**Published:** 2024-12-19

**Authors:** Kaiyuan Shang, Jiuqiang Guan, Tianwu An, Hongwen Zhao, Qin Bai, Huade Li, Quan Sha, Mingfeng Jiang, Xiangfei Zhang, Xiaolin Luo

**Affiliations:** ^1^Sichuan Academy of Grassland Sciences, Chengdu, China; ^2^College of Animal and Veterinary Sciences, Southwest Minzu University, Chengdu, China

**Keywords:** perinatal period, yak, nutritional supplementation, serum biochemistry, metabolomics

## Abstract

The transition period is a crucial stage in the reproductive cycle for dams and is linked closely with postpartum recovery, reproduction performance, and health. The confronting problem in the yak industry is that transition yaks under a conventional grazing feeding regime endure nutritional deficiency since this period is in late winter and early spring of the Qinghai-Tibet Plateau with the lack of grass on natural pasture. Therefore, this study aimed to investigate the effects of perinatal nutritional supplementation and early weaning on serum biochemistry, reproductive performance, and metabolomics in transition yaks. Eighteen healthy yaks in late pregnancy (233.9 ± 18.3 kg, 2–4 parity) were randomly assigned to three groups: conventional grazing feeding (GF, *n* = 6), additional nutrition supplementation (SF, *n* = 6), and additional nutrition supplementation with early weaning (SW, *n* = 6). Yaks in the GF, SF, and SW groups were free grazing on the same pasture in the daytime from −30 to 90 d relative to parturition. Yaks in SF and SW groups received total mixed ration supplementation in the barn during the night throughout the trial. Calves in the SW group were early weaned and separated from the dam at 60 d postpartum. Maternal body weight was measured at −30 and 90 d, and serum samples were collected to analyze serum biochemistry, hormones, and metabolomics at −15, 30, and 90 d relative to calving. In the SF and SW groups, yaks showed significantly higher body weight gain, serum glucose, globulin, and total protein concentrations. Lipid transportation molecules apolipoprotein B100 and very low-density lipoprotein of SF and SW yaks were significantly increased along with the decreased lipid mobilization products non-esterified fatty acid and β-hydroxybutyric acid when compared to GF yaks at −15 and 30 d. At 90 d, serum non-esterified fatty acid and β-hydroxybutyric acid levels were significantly lower in SW yaks than in SF ones, while apolipoprotein B100 and very low-density lipoprotein levels were significantly higher in SW yaks than in GF yaks. The serum levels of metabolic regulatory hormones, including insulin, leptin, and insulin-like growth factor I were significantly increased, and glucagon was significantly reduced in the SF and SW groups than in the GF group at −15 and 30 d. Among serum reproductive hormones, SF and SW yaks had significantly higher estradiol and progesterone concentrations than GF ones at −15 and 30 d. Follicle-stimulating and luteinizing hormone levels were increased in SW group than in SF and GF ones at 90 d. The calving rates in the following year were 0% (GF), 16.7% (SF), and 83.3% (SW), respectively. The serum metabolomics analysis revealed 848 metabolites in positive mode and 350 in negative mode. With the perinatal nutritional supplementation, the lipid and energy metabolism of transition yaks were improved, meanwhile, lipid mobilization and estrogen production-related pathways were down-regulated. These data suggest that perinatal nutrition supplementation reduces body weight loss, improves glucose and lipid metabolic adaptation to the transition period, and improves yaks’ reproductive performance. Additionally, the combination of early weaning and nutritional supplementation results in lower lipid mobilization and up-regulation of lipid transportation and reproductive hormone secretion, which may further contribute to postpartum recovery and acceleration of the reproductive cycle.

## 1 Introduction

Yak (*Bos grunniens*) is a predominant livestock species on the Qinghai-Tibet Plateau, providing local herders with the necessary producing and living materials (1, 2). Additionally, yaks in conventional grazing systems experience severe forage shortage during the winter season, which lasts 6–8 months per year and potentially leads to 25% body weight loss or even death (3). Yaks generally come into heat in July–August when pasture becomes abundant, and their gestation period is approximately 259 d (4). This reproductive characteristic places the yak in the winter with forage deficiency throughout late gestation and early lactation. The late gestation period, when the fetus develops rapidly, is critical for the dam and fetus. During this stage, the imbalance between the nutrition requirement of fetal development and inadequate nutrient intake can result in several issues. These problems comprise mobilization of maternal body reserves, loss of body condition and subsequent fertility, insufficient nutrients supply for the fetus, and the reduction of birth weight of calf (5). After parturition, a large amount of nutrients are also needed for milk synthesis in contrast to the lack of grass ingestion (6). Moreover, most calves are breastfed with dams and naturally weaned at about one year of age. Thus, most yaks fail to initiate the subsequent estrus in the calving year, leading to low reproductive efficiency, with calving intervals typically extending to two or even three years (7).

The perinatal period refers to the physiological stage of pregnant female animals from 28 d before to 28 d after parturition (8). This period is a crucial transitional phase during pregnancy that significantly influences fetal development, maternal health, and reproductive performance (9, 10). Research on dairy cows (11) has established that the demand for energy in the perinatal period increased dramatically due to the needs of pregnancy and lactation, while the dry matter intake of cows during this period is significantly reduced, resulting in an obstacle to energy supply. The dam in a negative energy balance (NEB) has to mobilize body reserve by lipid catabolism for maintenance metabolism, pregnancy, and lactation, which may produce plenty of ketone bodies and even cause ketosis. Previous reports (12, 13) on beef cattle demonstrated that early-weaned cows exhibit shorter postpartum intervals and higher conception rates than lactating cows. However, there is limited research on the effects of periparturient nutrition manipulation and early weaning on metabolic adaptation to the perinatal period and reproductive performance of yaks, which needs to be further investigated.

It was reported that serum metabolomics could be an effective research method for exploring the transition of nutrient metabolism in periparturient females (14, 15). However, metabolomics has barely been utilized to analyze the nutritional metabolism of perinatal yaks. This study assessed the impacts of nutritional supplementation and early weaning on maternal body weight change, serum biochemistry, hormone secretion, and serum metabolome in perinatal yaks, to explore feasible perinatal strategies to improve metabolic transition and reproductive performance.

## 2 Materials and methods

### 2.1 Experimental design and feeding management

The animal experiment of this research was approved by the Experimental Animal Ethics Committee of the Sichuan Academy of Grassland Sciences (Approval No. 20220012). It consisted of an adapting period of 10 days and an experimental period of 120 days. Eighteen healthy yaks in late-gestation (body weight 233.9 ± 18.3 kg, 2–4 parity) diagnosed with single calf were selected and randomly divided into three groups (1) conventional grazing feeding (GF), (2) grazing feeding with nutritional supplementation group (SF) and (3) grazing feeding with nutritional supplementation + early weaning group (SW), with six replicates (*n* = 6) in each group. Yaks were enrolled at −50 d before the expected parturition. Yaks in the GF, SF, and SW groups were grazing on the same grassland under the same management from −30 to 90 d relative to parturition, being released to pasture at 08:00 and returning to the barn at 18:00 daily. They were kept in individual stalls during the night (18:00–08:00). Yaks of SF and SW groups received total mixed ration (TMR) supplementation in the barn after daily grazing from −30 to 90 d. Calves were breastfed and naturally weaned postpartum without intervention for GF and SF yaks, while calves in the SW group were early weaned and separated from the dam at 60 d postpartum.

The supplemental TMR rations for SF and SW groups were formulated based on the estimation of grazing nutrients intake of experimental yaks in preliminary work, and to make up the gap between grazing ingestion and nutrition requirement according to the nutrition requirements of late pregnant and early lactating cattle in the “Feeding Standard of Beef Cattle in China (2004).” The ratio of concentrate to roughage of TMR was 45:55 in late gestation and 50:50 in early lactation, respectively (Table 1). The active dry yeast and functional peptides were purchased from Angel Yeast Co., Ltd. and Chengdu Mytech Biotech Co., Ltd., China. The nutrient levels of the milk replacer and starter diets for early-weaned calves are shown in Supplementary Table S1.

**Table 1:** Animal diet of supplemental feeding groups (DM basis).

Ingredients (%)	Late pregnancy diet	Early lactation diet^a^
Oat hay	55.00	50.00
Corn	21.55	27.82
Wheat bran	12.08	5.95
Soybean meal	2.13	4.01
Rapeseed meal	2.75	2.94
Cottonseed meal	1.37	1.73
Fermentation concentrates	2.29	3.05
Rumen-protected fat	-	1.09
Active dry yeast	0.13	0.14
CaHPO_4_	0.62	0.87
NaHCO_3_	0.57	0.74
NaCl	0.51	0.57
Functional peptides	0.49	—
Rumen-protected glucose	—	0.52
Premix^b^	0.51	0.57
Nutrient levels^c^		
NEmf (MJ/kg)	4.81	5.32
CP (%)	11.71	11.98
NDF (%)	46.9	41.96
ADF (%)	27.93	25.9
Ca (%)	1.29	1.37
P (%)	0.58	0.57

a The late pregnancy diet and early lactation diet were fed to the yaks in SF and SW groups from −30 d to parturition and from parturition to 90 d postpartum, respectively.

b The per kg premix provided the following: VA 2,750,000 IU, VD_3_ 750,000 IU, VE 14,500 IU, Cu 6,000 mg, Mn 20,000 mg, Zn 25,000 mg, I 240 mg, Se 140 mg, Co 190 mg.

c CP, crude protein; EE, ether extract; NDF, neutral detergent fiber; ADF, acid detergent fiber; Ca, calcium; P, phosphorus.

### 2.2 Sample collection and analysis

#### 2.2.1 Production performance

The body weight of the experimental yaks were measured before morning feeding at the beginning of the trial and 90 d after calving to calculate the body weight change. The feed intake of TMR was recorded daily, and the feed amount offered to each yak was determined by the feed refusal of the previous day to target <10% refusal.

#### 2.2.2 Serum biochemical

Blood samples were collected from the jugular vein of each yak into vacuum tubes before morning grazing (08:00) every 15 d until parturition in the prenatal stage. Samples close to −15 d relative to the actual calving date were selected as prepartum samples. Blood was harvested at 30 and 90 d after parturition during the postpartum stage. These samples were centrifuged at 3,500 rpm for 10 min to obtain serum, and aliquots of serum were stored at −80°C for subsequent analysis.

Glucose (GLU), cholesterol (CHO), triglyceride (TG), albumin (ALB), and total protein (TP) in serum samples at −15, 30, and 90 d were determined by an automatic biochemistry analyzer (BS360S, Shenzhen Mindray Co., Ltd., China) using corresponding reagents (Shenzhen Mindray Co., Ltd., China). Globulin (GLB) concentration was calculated as TP minus ALB. Enzyme-linked immunosorbent assay (ELISA) kits (Shanghai Enzyme-Linked Biotechnology Co., Ltd., China) were used to measure the serum apolipoprotein B100 (APOB100), very low-density lipoprotein (VLDL), and β-hydroxybutyric acid (BHBA) levels with a 96-well plate and microplate spectrophotometer (μQuant, BioTek Instruments, Inc., United States). Meanwhile, serum nonesterified fatty acid (NEFA) was analyzed using a commercial kit (E-BC-K013-M, Shanghai Elabscience Biotechnology Co., Ltd., China).

#### 2.2.3 Metabolic regulation hormones

The measurements of serum insulin (INS), glucagon (GC), insulin-like growth factor I (IGF-I), and leptin (LEP) concentrations were also performed using commercial ELISA kits (Shanghai Elabscience Biotechnology Co., Ltd., China) with the same instrument.

#### 2.2.4 Reproduction hormone and performance

The secretion levels of reproduction hormones, including estradiol (E_2_), follicle-stimulating hormone (FSH), luteinizing hormone (LH), and progesterone (PROG), were measured in serum samples at −15, 30, and 90 d with ELISA kits purchased from the same company. The calving rate in the following year was recorded for all yaks.

### 2.3 Statistical analysis

Yaks were excluded from the trial if they received nutritional supplementation less than 30 days before parturition to ensure the nutrition supply covered the entire perinatal period. The experimental data, except metabolomics, were statistically analyzed using the general linear model (GLM) of SAS 9.4 (SAS Institute, Inc.). Student–Newman–Keuls (SNK) statements were constructed to compare experimental treatments (nutritional supplementation and early weaning). Significance was declared at *p* ≤ 0.05, and trends were discussed when 0.05 < *p* ≤ 0.10.

### 2.4 Metabolomics analysis

#### 2.4.1 LC-MS untargeted metabolomics procedures and parameters

Serum samples from all groups at −15, 30, and 90 d were selected for Liquid Chromatograph Mass Spectrometer (LC-MS) untargeted metabolomics analysis (Beijing Novogene Co., Ltd., China). After sample pretreatment, the supernatant was collected and injected for LC-MS analysis (16–18). The experimental parameters were as follows:

Chromatographic conditions: Hypesil Gold column (C18), column temperature of 40°C, injection flow rate of 0.2 mL/min, positive mode (mobile phase A: 0.1% formic acid, mobile phase B: methanol), and negative mode (mobile phase A: 5 mM ammonium acetate, pH 9.0 mobile phase B: methanol).

Mass spectrometry conditions: The scanning range was set from m/z 100 to 1,500. The ESI source settings were spray voltage: 3.5 kV, sheath gas flow rate: 35 psi, aux gas flow rate: 10 L/min, Capillary Temp: 320°C, S-lens RF level: 60, and Aux gas heater temperature: 350°C. Polarity was set to positive and negative. MS/MS secondary scans were conducted in a data-dependent manner.

#### 2.4.2 Metabolomics data processing and statistical analyses

The metabolites were annotated using the Kyoto Encyclopedia of Genes and Genomes (KEGG) database,^1^ the HMDB database,^2^ and the LIPID Maps database.^3^ Multivariate statistical analyses were conducted with metabolomics data processing software metaX (19). Data after conversion underwent principal component analysis (PCA) and partial least squares discriminant analysis (PLS-DA) to derive the variable importance in projection (VIP) values for each metabolite. The T-test was performed for statistical significance (*p*-value) and fold change (FC) between groups. Correlation analysis (Pearson coefficient) among metabolites and statistical significance were analyzed using R programming language. Statistical significance was defined as *p* < 0.05. Visualization utilized R’s corrplot package for volcano plots and bubble plots.

## 3 Results

### 3.1 Production performance

There was no significant difference in the initial body weight of the yaks among the three groups (*p* > 0.05, Table 2). Significant treatment effects on body weight change were observed (*p* < 0.05), showing values of 1.58 kg, 14.00 kg, and 20.67 kg for GF, SF, and SW yaks, respectively. Additionally, the dry matter intake (DMI) of yaks between the SF and SW groups did not differ significantly (*p* > 0.05).

**Table 2:** Effects of perinatal nutritional supplementation and early weaning on production performance of yaks.

Item	Group^1^			SEM	*p*-value^2^
	GF	SF	SW		
Initial body weight (kg)	236.50	234.08	231.25	7.55	0.895
Postpartum 90 d final weight (kg)	238.08	248.08	251.92	8.46	0.539
Body weight change (kg)^2^	1.58^c^	14.00^b^	20.67^a^	1.97	<0.001
Dry matter intake of supplemental feeding (kg)	—	4.05	3.98	0.03	0.127

1 Yaks in the GF, SF and SW groups were free grazing on the same pasture from −30 to 90 d relative to parturition, being released to pasture at 08:00 and returning to barn at 18:00. Yaks in SF and SW groups received total mixed ration supplementation in barn during the night (18:00–08:00) from −30 to 90 d. Calves in the SW group were early weaned and separated from the dam at 60 d postpartum.

2 Values with different superscript letters in the same row indicate significant differences (*p* < 0.05), and the same superscript letter or no letter indicates no significant difference (*p* > 0.05). The same as below.

### 3.2 Serum biochemistry

Regarding serum metabolites associated with glucose and nitrogen metabolism (Table 3), GLU and GLB levels were significantly elevated in SF and SW yaks at −15 and 30 d (*p* < 0.05). ALB levels did not differ significantly among treatments (*p* > 0.05). TP levels were significantly higher in the yaks received SF and SW treatments at 30 d than in the GF yaks (*p* < 0.05). GLU concentration exhibited significant differences between the SW and GF yaks at 90 d (*p* < 0.05), with nonsignificant differences between SF and SW.

**Table 3:** Effects of perinatal nutritional supplementation and early weaning on serum glucose and nitrogen metabolism related metabolites in yaks.

Item^2^	Day relative to calving	Group^1^			SEM	*p*-value
		GF	SF	SW		
GLU (mmol/L)	−15	2.04^b^	2.98^a^	3.07^a^	0.20	0.005
	30	1.90^b^	2.67^a^	2.63^a^	0.14	0.002
	90	1.76^b^	2.52^ab^	3.21^a^	0.24	0.004
ALB (g/L)	−15	21.97	21.98	22.13	2.29	0.999
	30	19.37	22.18	21.57	1.46	0.396
	90	21.55	22.52	22.48	1.35	0.857
GLB (g/L)	−15	29.10^b^	34.68^a^	34.00^a^	1.55	0.047
	30	22.33^b^	27.63^a^	27.05^a^	1.48	0.050
	90	22.47	23.82	20.15	0.97	0.060
TP (g/L)	−15	51.07	56.67	56.13	3.22	0.432
	30	41.70^b^	49.82^a^	48.62^a^	2.20	0.042
	90	44.02	46.33	42.63	1.74	0.375

1 Yaks in the GF, SF and SW groups were free grazing on the same pasture from −30 to 90 d relative to parturition, being released to pasture at 08:00 and returning to barn at 18:00. Yaks in SF and SW groups received total mixed ration supplementation in barn during the night (18:00–08:00) from −30 to 90 d. Calves in the SW group were early weaned and separated from the dam at 60 d postpartum.

2 GLU, glucose; ALB, albumin; GLB, globulin; TP, total protein.

Among the serum metabolites related to lipid metabolism (Table 4), serum TG levels significantly decreased in the yaks of the SF and SW groups at 30 d compared to those of GF (*p* < 0.05). Significantly increased prepartum and postpartum CHO concentrations in response to SF and SW treatments were observed (*p* < 0.05). Yaks in the SF and SW groups had significantly lower serum NFEA and BHBA levels than those in the GF group (*p* < 0.05), and there was a further significant reduction with SW yaks (*p* < 0.05). APOB100 and VLDL concentrations were significantly increased at −15 and 30 d (*p* < 0.05) in SF and SW yaks compared with GF ones. Furthermore, the differences in serum APOB100 and VLDL levels between SW and GF groups reached significant levels at 90 d postpartum (*p* < 0.05).

**Table 4:** Effects of perinatal nutritional supplementation and early weaning on serum lipid metabolism related metabolites of yaks.

Item^2^	Day relative to calving	Group^1^			SEM	*p*-value
		GF	SF	SW		
TG (mmol/L)	−15	0.2	0.24	0.23	0.02	0.538
	30	0.19^a^	0.11^b^	0.12^b^	0.02	0.013
	90	0.15	0.12	0.12	0.01	0.305
CHO (mmol/L)	−15	1.10^b^	1.53^a^	1.53^a^	0.1	0.015
	30	0.79^b^	1.07^a^	1.12^a^	0.09	0.034
	90	1.05^b^	1.38^ab^	1.73^a^	0.11	0.004
NEFA (mmol/L)	−15	1.50^a^	1.21^b^	1.20^b^	0.02	<0.001
	30	1.68^a^	1.40^b^	1.40^b^	0.02	<0.001
	90	1.59^a^	1.43^b^	1.28^c^	0.03	<0.001
BHBA (μmol/L)	−15	469.63^a^	337.22^b^	307.40^b^	26.55	0.003
	30	836.51^a^	553.39^b^	532.95^b^	30.29	<0.001
	90	636.55^a^	463.55^b^	283.38^c^	26.05	<0.001
VLDL (mmol/L)	−15	26.43^b^	31.81^a^	31.21^a^	0.91	0.002
	30	20.48^b^	25.02^a^	26.04^a^	1.05	0.005
	90	21.86^b^	25.47^ab^	29.00^a^	1.4	0.010
APOB100 (μg/mL)	−15	31.90^b^	42.49^a^	41.03^a^	1.82	0.002
	30	21.86^b^	29.75^a^	29.91^a^	1.4	0.002
	90	22.78^b^	26.48^b^	34.08^a^	1.73	0.002

1 Yaks in the GF, SF and SW groups were free grazing on the same pasture from −30 to 90 d relative to parturition, being released to pasture at 08:00 and returning to barn at 18:00. Yaks in SF and SW groups received total mixed ration supplementation in barn during the night (18:00–08:00) from −30 to 90 d. Calves in the SW group were early weaned and separated from the dam at 60 d postpartum.

2 TG, triglyceride; CHO, cholesterol; NEFA, nonesterified fatty acid; BHBA, β-hydroxybutyric acid; VLDL, very low-density lipoprotein; APOB100, apolipoprotein B100.

### 3.3 Metabolic regulation hormones

As for hormones associated with metabolic regulation (Table 5), serum INS, LEP, and IGF-I levels significantly increased in the SF and SW groups at −15 and 30 d (*p* < 0.05). In contrast, SF and SW yaks had significantly lower GC concentrations than GF ones (*p* < 0.05). At 90 d, serum INS and IGF-I levels were significantly higher in the SW group than in the GF (*p* < 0.05).

**Table 5:** Effects of perinatal nutritional supplementation and early weaning on serum hormones related to metabolism regulation in yaks.

Item^2^	Day relative to calving	Group^1^			SEM	*p*-value
		GF	SF	SW		
INS (mIU/L)	−15	35.95^b^	42.35^a^	43.72^a^	1.24	0.001
	30	30.62^b^	37.32^a^	38.50^a^	1.74	0.013
	90	28.27^b^	32.62^ab^	36.06^a^	1.45	0.010
GC (pg/mL)	−15	226.30^a^	188.37^b^	184.26^b^	9.36	0.014
	30	254.49^a^	197.88^b^	202.48^b^	8.9	0.001
	90	194.92	187.88	180.00	10.59	0.628
LEP (ng/mL)	−15	4.20^b^	5.62^a^	5.35^a^	0.3	0.017
	30	4.07^b^	5.04^a^	5.10^a^	0.28	0.040
	90	4.97	5.25	6.29	0.33	0.053
IGF-I (ng/mL)	−15	202.31^b^	280.79^a^	285.24^a^	11.64	<0.001
	30	161.42^b^	222.65^a^	223.78^a^	13.02	0.007
	90	199.62^b^	235.29^b^	285.29^a^	12.85	0.002

1 Yaks in the GF, SF and SW groups were free grazing on the same pasture from −30 to 90 d relative to parturition, being released to pasture at 08:00 and returning to barn at 18:00. Yaks in SF and SW groups received total mixed ration supplementation in barn during the night (18:00–08:00) from −30 to 90 d. Calves in the SW group were early weaned and separated from the dam at 60 d postpartum.

2 INS, insulin; GC, glucagon; LEP, leptin; IGF-I, insulin-like factor I.

### 3.4 Reproductive hormones and reproduction rate for the next year

Significantly increased serum E_2_ and PROG concentrations in response to SF and SW treatments were observed at −15 and 30 d (Table 6, *p* < 0.05). At 90 d postpartum, SW yaks had significantly greater E_2_ and PROG levels than GF yaks (*p* < 0.05). With no difference among treatments at −15 and 30 d, the serum FSH and LH of yaks receiving SW treatment were elevated significantly compared to GF and SF at 90 d. Yaks in the GF, SF, and SW groups had calving rates of 0, 16.7, and 83.3% in the following year, respectively (Table 7).

**Table 6:** Effects of perinatal nutritional supplementation and early weaning on serum reproductive hormones of yaks.

Item^2^	Day relative to calving	Group^1^			SEM	*p*-value
		GF	SF	SW		
FSH (mIU/mL)	−15	2.71	3.14	3.03	0.4	0.781
	30	6.00	5.75	6.03	0.45	0.895
	90	8.01^c^	9.65^b^	11.45^a^	0.47	0.001
LH (mIU/mL)	−15	4.33	4.66	4.59	0.43	0.855
	30	6.94	7.45	7.58	0.49	0.636
	90	12.07^b^	13.01^b^	15.05^a^	0.6	0.011
E_2_ (pg/mL)	−15	53.54^b^	74.32^a^	72.53^a^	3.41	0.001
	30	17.99^b^	24.79^a^	27.46^a^	2.02	0.016
	90	24.40^b^	29.37^ab^	35.13^a^	2.78	0.049
PROG (ng/mL)	−15	42.28^b^	53.68^a^	54.08^a^	1.85	<0.001
	30	15.43^b^	23.41^a^	24.91^a^	1.35	<0.001
	90	23.71^b^	25.35^b^	33.24^a^	1.28	<0.001

1 Yaks in the GF, SF and SW groups were free grazing on the same pasture from −30 to 90 d relative to parturition, being released to pasture at 08:00 and returning to barn at 18:00. Yaks in SF and SW groups received total mixed ration supplementation in barn during the night (18:00–08:00) from −30 to 90 d. Calves in the SW group were early weaned and separated from the dam at 60 d postpartum.

2 FSH, follicle stimulating hormone; LH, luteinizing hormone; E_2_, estradiol; PROG, progesterone.

**Table 7:** Effect of perinatal nutritional supplementation and early weaning on the calving rate of yaks in the following year.

Item	Group^a^		
	GF	SF	SW
Next-year calving rate (%)	0	16.7	83.3

a Yaks in the GF, SF and SW groups were free grazing on the same pasture from −30 to 90 d relative to parturition, being released to pasture at 08:00 and returning to barn at 18:00. Yaks in SF and SW groups received total mixed ration supplementation in barn during the night (18:00–08:00) from −30 to 90 d. Calves in the SW group were early weaned and separated from the dam at 60 d postpartum.

### 3.5 Serum metabolomics analysis

#### 3.5.1 PLS-DA analysis

**Figure 1:**
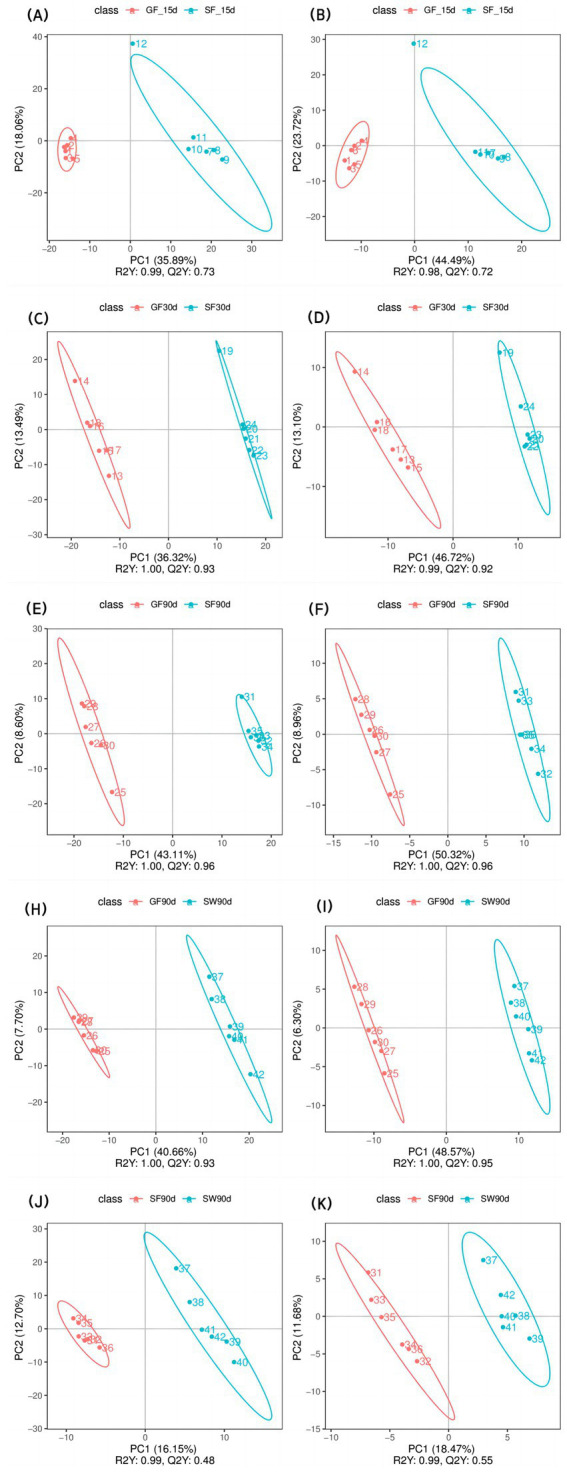
PLS-DA analysis of the effects of perinatal nutritional supplementation and early weaning on serum metabolomics in yaks. Yaks in the GF, SF and SW groups were free grazing on the same pasture from −30 to 90 d relative to parturition, being released to pasture at 08:00 and returning to barn at 18:00. Yaks in SF and SW groups received total mixed ration supplementation in barn during the night (18:00–08:00) from −30 to 90 d. Calves in the SW group were early weaned and separated from the dam at 60 d postpartum. (A,B) SF vs. GF in positive and negative modes at −15 d, (C,D) SF vs. GF in positive and negative modes at 30 d, (E,F) SF vs. GF in positive and negative modes at 90 d. (G,H) SW vs. GF in positive and negative modes at 90 d. (I,J) SW vs. SF in positive and negative modes at 90 d.

The PLS-DA scatter plots of serum samples (Figure 1) exhibited high sample aggregation within each treatment group in positive and negative modes, indicating good repeatability. Additionally, differences among the groups were observed, suggesting that different experimental treatments significantly influenced the serum metabolome of periparturient yaks. In all datasets, R^2^Y > Q^2^Y indicated a well-established model (20).

#### 3.5.2 Results of differential metabolite screening

Thresholds were set at VIP >1.0, FC >1.2 or FC <0.833, and *p*-value <0.05 (21–23), and the number of differential metabolites screened is listed in Supplementary Figure S1 and Supplementary Table S2. Eight hundred forty-eight and Three hundred fifty significantly differential metabolites were identified in the positive and negative modes, respectively.

#### 3.5.3 Analysis of the top 20 differential metabolites

**Figure 2:**
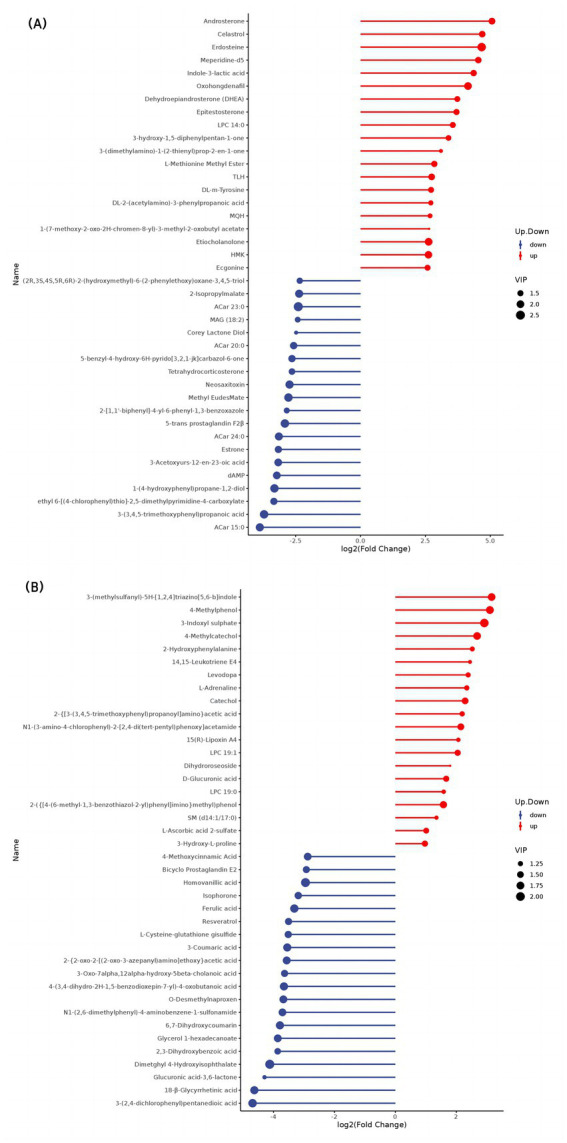
Stem map of the effects of perinatal nutritional supplementation and early weaning on serum metabolomics at −15 d relative to parturition. Yaks in the GF, SF and SW groups were free grazing on the same pasture from −30 to 90 d relative to parturition, being released to pasture at 08:00 and returning to barn at 18:00. Yaks in SF and SW groups received total mixed ration supplementation in barn during the night (18:00–08:00) from −30 to 90 d. Calves in the SW group were early weaned and separated from the dam at 60 d postpartum. (A,B) SF vs. GF in positive and negative modes at −15 d.

**Figure 3:**
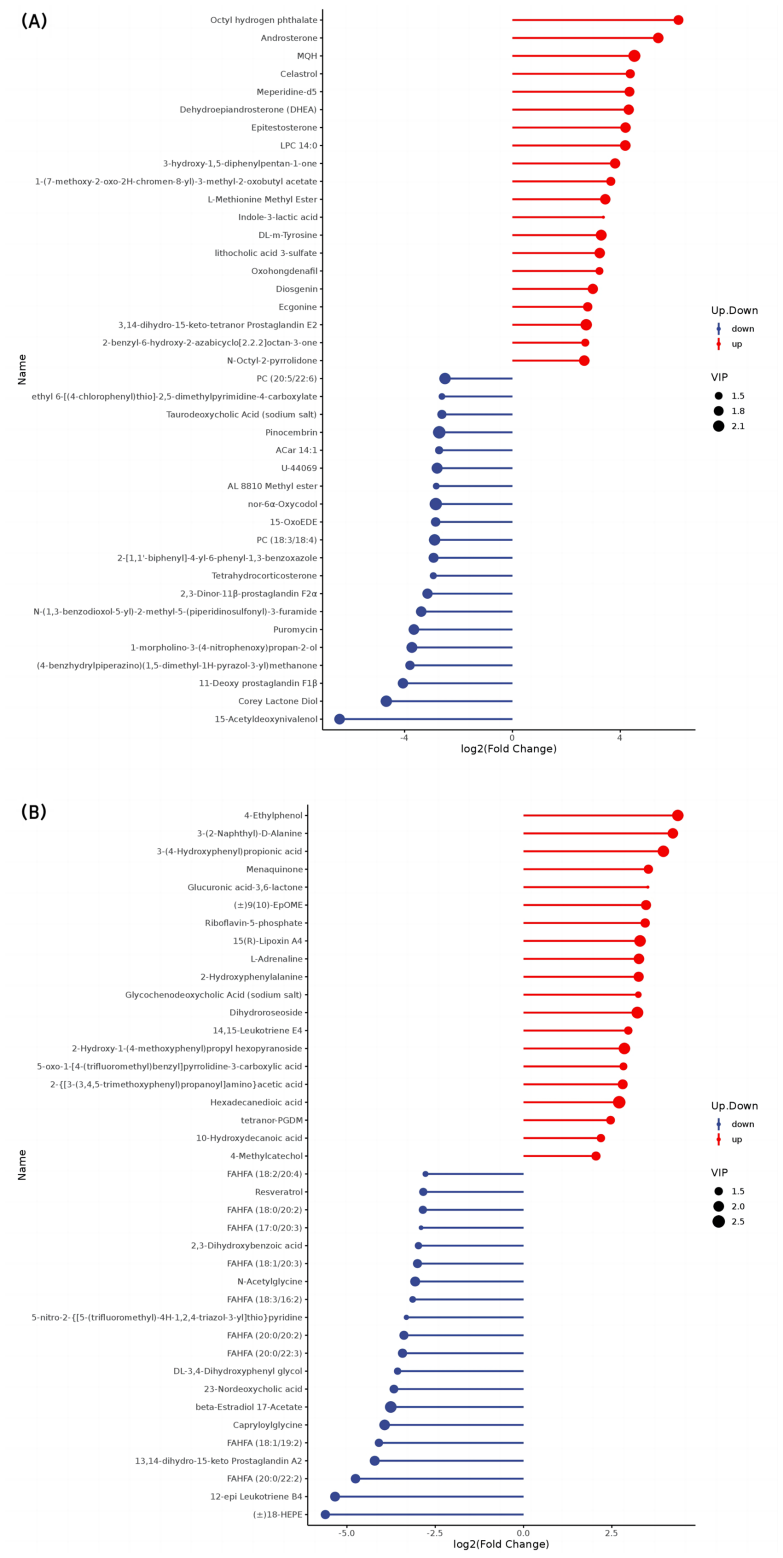
Stem map of the effects of perinatal nutritional supplementation and early weaning on serum metabolomics at 30 d relative to parturition. Yaks in the GF, SF and SW groups were free grazing on the same pasture from −30 to 90 d relative to parturition, being released to pasture at 08:00 and returning to barn at 18:00. Yaks in SF and SW groups received total mixed ration supplementation in barn during the night (18:00–08:00) from −30 to 90 d. Calves in the SW group were early weaned and separated from the dam at 60 d postpartum. (A,B) SF vs. GF in positive and negative modes at 30 d.

**Figure 4:**
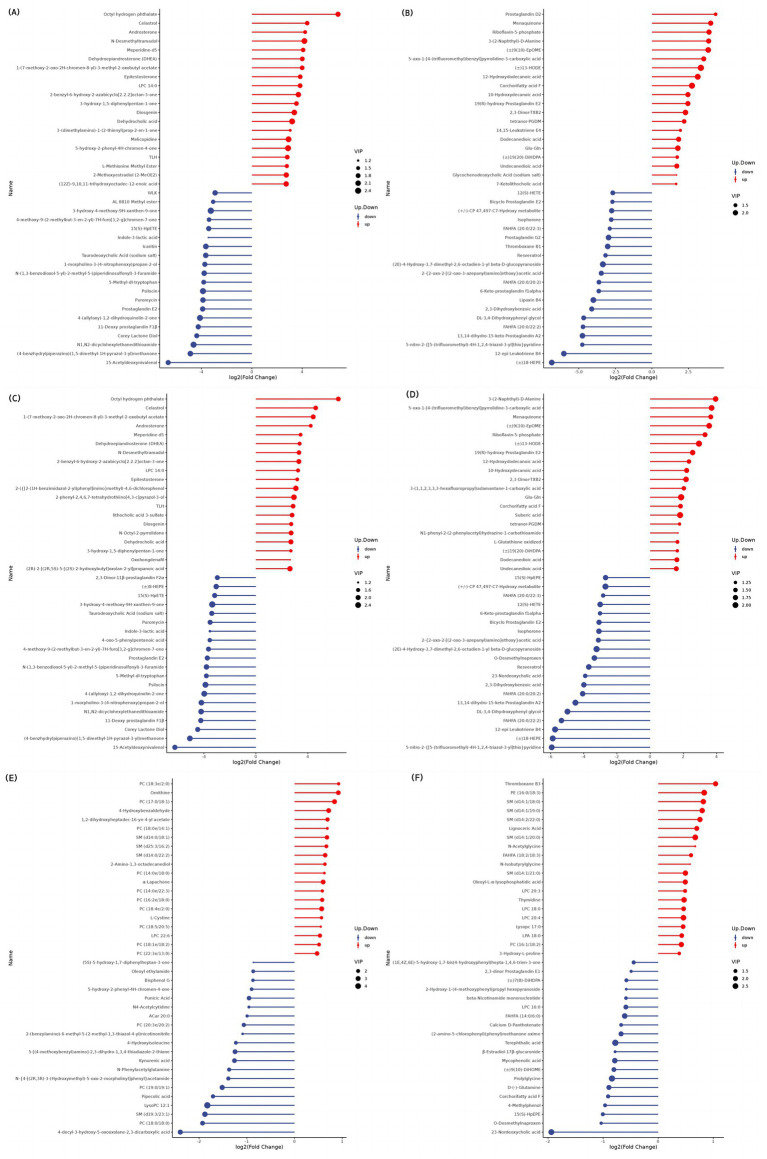
Stem map of the effects of perinatal nutritional supplementation and early weaning on serum metabolomics at 90 d relative to parturition. Yaks in the GF, SF and SW groups were free grazing on the same pasture from −30 to 90 d relative to parturition, being released to pasture at 08:00 and returning to barn at 18:00. Yaks in SF and SW groups received total mixed ration supplementation in barn during the night (18:00–08:00) from −30 to 90 d. Calves in the SW group were early weaned and separated from the dam at 60 d postpartum. (A,B) SF vs. GF in positive and negative modes at 90 d. (C,D) SW vs. GF in positive and negative modes at 90 d. (E,F) SW vs. SF in positive and negative modes at 90 d.

The top 20 differential metabolites in the pairwise comparison of all treatments are illustrated in Figures 2–4.

Results showed an increase in lysphosphatidylcholine (LPC) (14:0, 19:0, 19:1) concentrations and decreased 3-acetoxyurs-12-en-23-oic acid, 2-isopropylmalate, and glycerol 1-hexadecanoate levels (*p* < 0.05) in SF yaks compared to GF yaks at −15 d. SF yaks had higher serum DL-m-tyrosine, 2-hydroxyphenylalanine, and 3-hydroxy-L-proline concentrations (*p* < 0.05) in amino acid metabolism than GF. Regarding the organic acid metabolism, 3-indoxyl sulfate and D-glucuronic acid concentrations significantly increased and reduced methyl eudesmate levels (*p* < 0.05) in the SF group than in GF. SF yaks reached a significant decrease in the levels of deoxyadenosine monophosphate (dAMP) (*p* < 0.05), an indicator of nucleotide metabolism. SF yaks had significantly elevated 15(R)-Lipoxin A4, androsterone, dehydroepiandrosterone, epitestosterone, and etiocholanolone levels, while estrone, 5-trans prostaglandin F2β, and bicyclo prostaglandin E2 concentrations were notably reduced (*p* < 0.05) in hormone and hormone derivative metabolism.

At 30 d, SF yaks had higher LPC 14:0, hexadecanedioic acid, and 10-hydroxydecanoic acid levels (*p* < 0.05), and lower phosphatidylcholine (PC) [(18:3/18:4), (20:5/22:6)], branched fatty acid esters of hydroxy fatty acids (FAHFAs) [(20:0/22:2), (18:1/19:2), (20:0/22:3), (20:0/20:2), (18:3/16:2), (18:1/20:3), (17:0/20:3), (18:0/20:2), (18:2/20:4)], and 15-Acetyldeoxynivalenol concentrations related to lipid metabolism compared to GF yaks (*p* < 0.05). Regarding amino acid metabolism, SF yaks had elevated DL-m-tyrosine and 2-hydroxyphenylalanine levels (*p* < 0.05) but lower L-(+)-citrulline, gamma-glutamylleucine, homoarginine, cinnamoylglycine, capryloylglycine, and N-acetyl glycine levels (*p* < 0.05). With respect to organic acid metabolism, SF yaks showed higher tetranor-PGDM and oxohongdenafil levels than GF (*p* < 0.05). Additionally, riboflavin-5-phosphate concentrations were higher in the SF group (*p* < 0.05), while puromycin levels decreased (*p* < 0.05) involved in nucleotide metabolism. SF yaks had significantly higher 15(R)-lipoxin A4, androstenedione, dehydroepiandrosterone, and epitestosterone concentrations (*p* < 0.05) but lower 11-deoxy prostaglandin F1β, 2,3-dinor-11β-prostaglandin F2α, and 13,14-dihydro-15-keto prostaglandin A2 levels (*p* < 0.05) in hormone and hormone derivative metabolism than GF ones. Lastly, vitamin K2 levels were significantly higher in the SF group (*p* < 0.05).

At 90 d, regarding lipid metabolism, SF yaks showed significant increases in LPC 14:0, corchorifatty acid F, dodecanedioic acid, undecanedioic acid, 12-hydroxydodecanoic acid, and 10-hydroxydecanoic acid concentrations (*p* < 0.05), meanwhile, significant reduction in 15(S)-HpETE, FAHFAs [(20:0/20:2), (20:0/22:2), (20:0/22:3)], and 12(S)-HpETE levels when compared to the GF group (*p* < 0.05). Glu-Gln and L-Glutathione oxidized concentrations increased (*p* < 0.05) in the SF group in amino acid metabolism. SF yaks had elevated 2,3-dinor-TXB2 and tetranor-PGDM levels (*p* < 0.05) related to organic acid metabolism. Additionally, riboflavin-5-phosphate concentrations were higher (*p* < 0.05), while puromycin levels were lower (*p* < 0.05) in the SF group in nucleotide metabolism. In hormone and hormone derivative metabolism, the SF group showed a significant effect on higher androstenedione, dehydroepiandrosterone, epitestosterone, and 2-methoxyestradiol concentrations (*p* < 0.05) compared with the GF group, along with decreased levels of 11-deoxy prostaglandin F1β, 6-keto-prostaglandin f1alpha, prostaglandin E2, prostaglandin D2, 13,14-dihydro-15-keto prostaglandin A2, bicyclol prostaglandin E2, and lipoxin B4 (*p* < 0.05). Moreover, vitamin K2 levels were improved (*p* < 0.05) in the SF group in vitamin metabolism.

At 90 d, in the comparison between SW and GF groups, significant increases in serum LPC 14:0, corchorifatty acid F, suberic acid, dodecanedioic acid, undecanedioic acid, 12-hydroxydodecanoic acid, and 10-hydroxydecanoic acid concentrations involved in lipid metabolism (*p* < 0.05) were observed in SW group. In contrast, there were significant decreases in 15(S)-HETE, FAHFA [(20:0/22:2), (20:0/20:2), (20:0/22:3)], and 12(S)-HETE levels (*p* < 0.05). Glu-Gln and L-glutathione oxidized concentrations (*p* < 0.05) occurred with SW treatment in amino acid metabolism. SW yaks had significantly higher 2,3-Dinor-TXB2 and tetranor-PGDM levels (*p* < 0.05) related to organic acid metabolism. Additionally, riboflavin-5-phosphate levels increased (*p* < 0.05), while puromycin levels decreased (*p* < 0.05) regarding nucleotide metabolism in the SW group. With respect to hormone and hormone derivative metabolism, androsterone, dehydroepiandrosterone, and epitestosterone levels significantly increased (*p* < 0.05), and 11-deoxy prostaglandin F1β, prostaglandin E2, 2,3-dinor-11β-prostaglandin F2α, bicyclic prostaglandin E2, and 6-keto-prostaglandin f1alpha concentrations significantly decreased (*p* < 0.05) among yaks of SW group when compared with those of GF. Moreover, vitamin K2 levels were higher in the SW group (*p* < 0.05) than in the GF group.

At 90 d, serum PC [(18:3e/2:0), (17:0/18:1), (18:0e/14:1), (14:0e/18:0), (14:0e/22:3), (16:2e/18:0), (18:4e/2:0), (18:5/20:5), (18:1e/18:2), (22:3e/13:0)], LPC (12:1, 17:0, 22:6, 20:3, 18:0, 20:4), SM [(d14:0/18:1), (d25:3/16:2), (d14:0/22:2)], punicic acid, branched fatty acid esters of hydroxy fatty acids (FAHFAs) (18:2/18:3) and lysopc (12:1, 17:0) related to lipid metabolism levels were significantly elevated in SW yaks when compared to SF ones (*p* < 0.05). In contrast, octadecane tricarboxylic acid, oleoyl ethanamide, and 15(S)-HpEPE, corchorifatty acid F, LPC 16:0, pipecolic acid, and FAHFAs (14:0/6:0) concentrations were significantly lower (*p* < 0.05) in SW yaks. SW group had significantly increased ornithine, L-cystine, N-acetylglycine, N-isobutyrylglycine, and 3-hydroxy-L-proline concentrations (*p* < 0.05) among amino acid metabolites. Meanwhile, N-phenylacetylglutamine, 4-hydroxyisoleucine, D-(−)-glutamine, prolylglycine, and calcium D-panthotenate levels were significantly reduced by SW treatment (*p* < 0.05). Terephthalic acid concentration was significantly lower in the SW group (*p* < 0.05). In nucleotide metabolism, significantly higher thymidine levels (*p* < 0.05) were observed in SW yaks than in SF yaks. A significant reduction of N4-acetylcysteine and β-nicotinamide mononucleotide levels in serum of SW yaks was detected (*p* < 0.05). As for hormone and hormone derivative metabolism, decreased β-estradiol-17β-glucuronide levels (*p* < 0.05) were observed for SW yaks compared to SF ones.

#### 3.5.4 KEGG enrichment analysis

**Figure 5:**
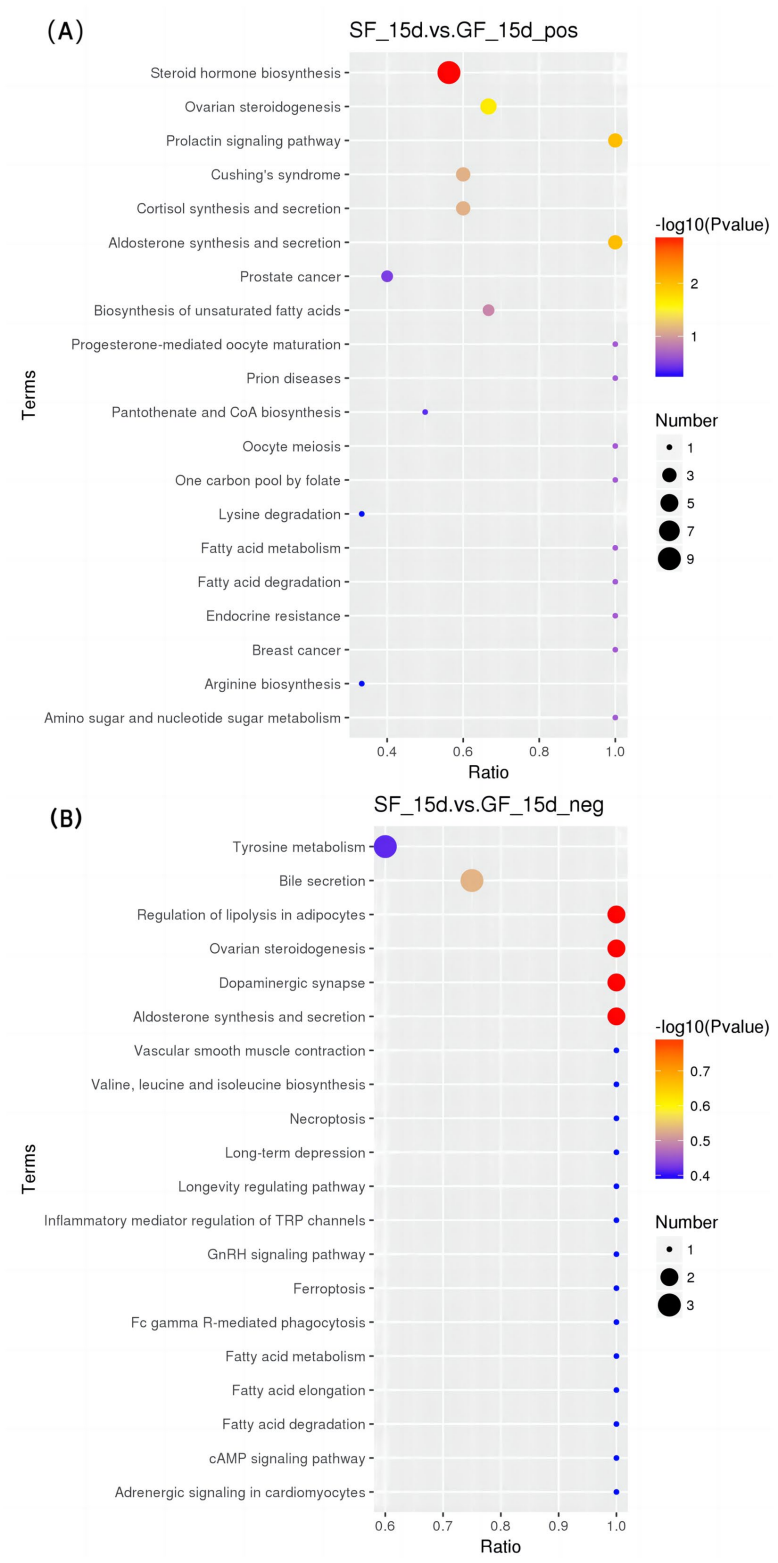
KEGG bubble diagram of the enriched pathways at −15 d relative to parturition. Yaks in the GF, SF and SW groups were free grazing on the same pasture from −30 to 90 d relative to parturition, being released to pasture at 08:00 and returning to barn at 18:00. Yaks in SF and SW groups received total mixed ration supplementation in barn during the night (18:00–08:00) from −30 to 90 d. Calves in the SW group were early weaned and separated from the dam at 60 d postpartum. (A,B) SF vs. GF in positive and negative modes at −15 d.

**Figure 6:**
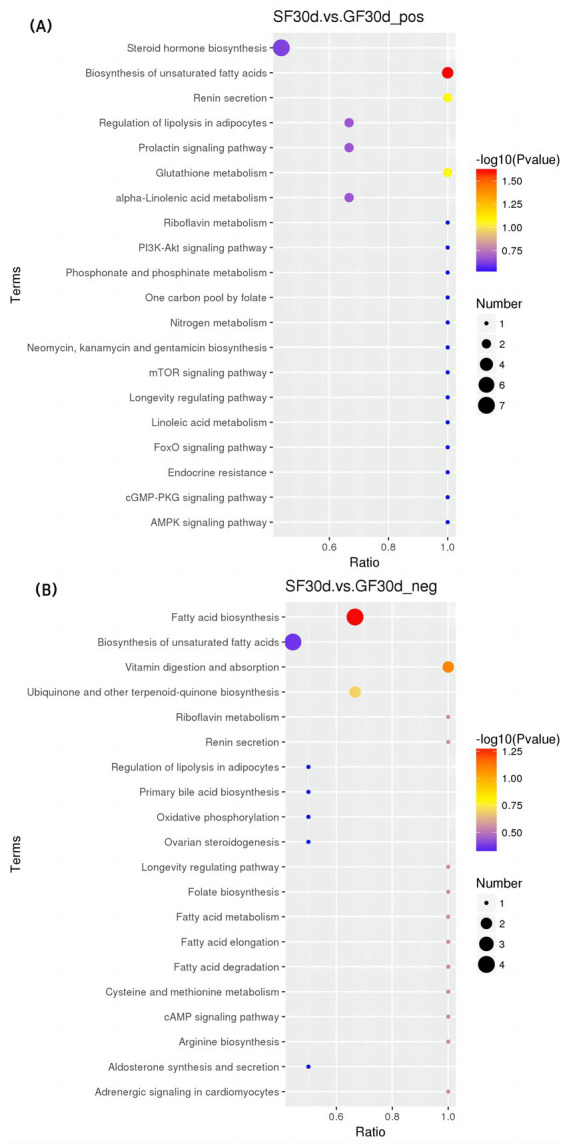
KEGG bubble diagram of the enriched pathways at 30 d relative to parturition. Yaks in the GF, SF and SW groups were free grazing on the same pasture from −30 to 90 d relative to parturition, being released to pasture at 08:00 and returning to barn at 18:00. Yaks in SF and SW groups received total mixed ration supplementation in barn during the night (18:00–08:00) from −30 to 90 d. Calves in the SW group were early weaned and separated from the dam at 60 d postpartum. (A,B) SF vs. GF in positive and negative modes at 30 d.

**Figure 7:**
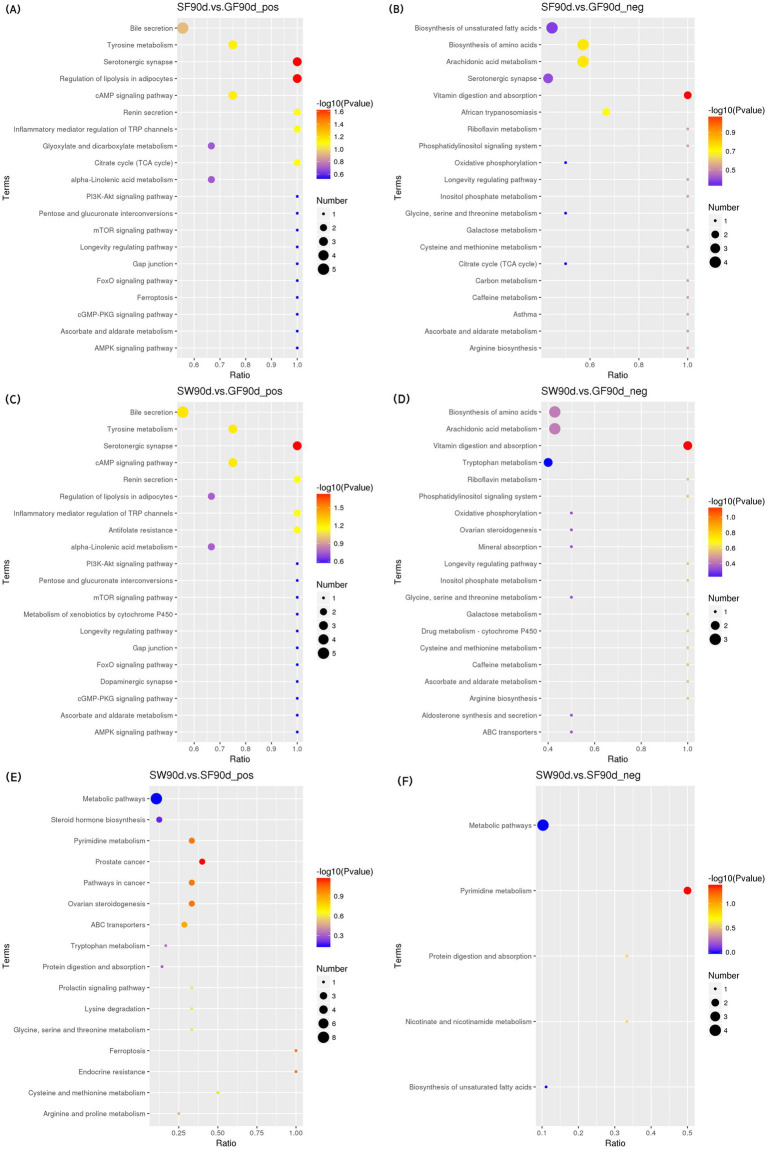
KEGG bubble diagram of the enriched pathways at 90 d relative to parturition. Yaks in the GF, SF and SW groups were free grazing on the same pasture from −30 to 90 d relative to parturition, being released to pasture at 08:00 and returning to barn at 18:00. Yaks in SF and SW groups received total mixed ration supplementation in barn during the night (18:00–08:00) from −30 to 90 d. Calves in the SW group were early weaned and separated from the dam at 60 d postpartum. (A,B) SF vs. GF in positive and negative modes at 90 d. (C,D) SW vs. GF in positive and negative modes at 90 d. (E,F) SW vs. SF in positive and negative modes at 90 d.

In the positive mode (Figures 5–7 and Table 8), four pathways were significantly enriched in the comparison between SF and GF groups at −15 d using KEGG, including steroid hormone biosynthesis [KEGG, map00140], prolactin signaling pathway [KEGG, map04917], aldosterone synthesis and secretion [KEGG, map04925], and ovarian steroidogenesis [KEGG, map04913]. In the steroid hormone biosynthesis pathway, androsterone and etiocholanolone were significantly increased in SF yaks (*p* < 0.05), and pregnenolone, cortodoxone, corticosterone, androstenedione, tetrahydrocorticosterone, estrone, and progesterone were significantly decreased (*p* < 0.05). In the prolactin signaling pathway, the SF treatment significantly reduced androstenedione, estrone, and progesterone levels (*p* < 0.05). The pregnenolone, corticosterone, and progesterone concentrations were significantly decreased in SF compared to GF yaks (*p* < 0.05) in the aldosterone synthesis and secretion pathway. In the ovarian steroidogenesis pathway, SF yaks had significantly decreased pregnenolone, androstenedione, estrone, and progesterone levels (*p* < 0.05).

**Table 8:** Significantly enriched metabolic pathways and significantly different metabolites in each pathway.

Item^a^	Mode	Metabolic pathways	Significantly different metabolites	*p*-value
SF15d vs. GF15d	Positive	Steroid hormone biosynthesis	Pregnenolone↓; Etiocholanolone↑; Cortodoxone↓; Corticosterone↓; Androsterone↑; Androstenedione↓; Tetrahydro corticosterone↓; Estrone↓; Progesterone↓	0.001
		Prolactin signaling pathway	Androstenedione↓; Estrone↓; Progesterone↓	0.010
		Aldosterone synthesis and secretion	Pregnenolone↓; Corticosterone↓; Progesterone↓	0.010
		Ovarian steroidogenesis	Pregnenolone↓; Androstenedione↓; Estrone↓; Progesterone↓	0.022
SF30d vs. GF30d	Positive	Biosynthesis of unsaturated fatty acids	Eicosapentaenoic acid↓; Docosapentaenoic acid↓; Docosahexaenoic acid↓	0.025
SF90d vs. GF90d	Positive	Serotonergic synapse	Serotonin↓; Prostaglandin E2↓; Prostaglandin J2↓	0.023
		Regulation of lipolysis in adipocytes	Corticosterone↓; Prostaglandin E2↓; Adenosine 5′-monophosphate↓	0.023
SW90d vs. GF90d	Positive	Serotonergic synapse	Serotonin↓; Prostaglandin E2↓; Prostaglandin J2↓	0.018
SW90d vs. SF90d	Negative	Pyrimidine metabolism	Thymidine↑; 2-Deoxyuridine↑	0.043

a Yaks in the GF, SF and SW groups were free grazing on the same pasture from −30 to 90 d relative to parturition, being released to pasture at 08:00 and returning to barn at 18:00. Yaks in SF and SW groups received total mixed ration supplementation in barn during the night (18:00–08:00) from −30 to 90 d. Calves in the SW group were early weaned and separated from the dam at 60 d postpartum.

At 30 d, SF yaks had one significantly enriched pathway for biosynthesis of unsaturated fatty acids [KEGG, map01040] in positive mode compared to GF groups. In this pathway, eicosapentaenoic, docosapentaenoic, and docosahexaenoic acid concentrations were significantly reduced in the SF group (*p* < 0.05).

At 90 d, two pathways were significantly enriched in the positive mode of the comparison between SF and GF groups, including serotonergic synapse [KEGG, map04726] and regulation of lipolysis in adipocytes [KEGG, map04923]. Significant reductions in serotonin (5-HT), prostaglandin E2, and prostaglandin J2 levels were observed in the serotonergic synaptic pathway in the comparison between the SF and GF groups (*p* < 0.05). Corticosterone, prostaglandin E2, and adenosine 5′-monophosphate concentration of SF yaks were significantly decreased in the regulation of lipolysis in the adipocytes pathway (*p* < 0.05).

One pathway significantly enriched in the positive mode of the comparison between the SW and GF groups at 90 d was the serotonergic synapse [KEGG, map04726], where the 5-HT, prostaglandin E2, and prostaglandin J2 metabolite concentrations were significantly decreased in SW yaks (*p* < 0.05). In the negative mode, the comparison between SW and SF groups at 90 d exhibited one significantly enriched pathway, that is, pyrimidine metabolism [KEGG, map00240], and we observed significantly increased thymidine and 2-deoxyuridine levels for SW yaks in this pathway (*p* < 0.05).

## 4 Discussion

In this study, we simulated the conventional grazing feeding regime of yaks by releasing all experimental yaks to pasture during the daytime (08:00–18:00) and keeping them in the barn at night (18:00–08:00) because this represents the typical and common grazing pattern on the Qinghai-Tibet Plateau (24). For a long time, in the conventional grazing regime, local herders let yaks graze daily and had to gather them back to the barn due to the cold temperature, frequent wolf attacks, and yak thieves at night. Under the same grazing condition, SF and SW groups were supplemented with additional TMR to compare adequate nutritional supply and inadequate nutrient ingestion in the conventional grazing of the GF group.

Body weight change is one of the most intuitive indicators for observing yaks’ nutritional status and production performance. In the present study, the body weight of yaks in the SF and SW groups increased by 14.00 kg and 20.67 kg, significantly higher than the 1.58 kg GF group. Studies on dairy cows (11) and ewes (25) have found that periparturient energy requirement rises dramatically due to the demands of pregnancy and lactation and that dams are very likely to suffer NEB, leading to significant body weight loss. In that case, sufficient nutrient intake during the perinatal period can alleviate the weight loss. Lusby et al. (26) reported that cows gained more weight in the summer after early weaning. Our results also revealed that adequate nutrition supplementation during the perinatal period and early weaning strategies for yaks were conducive to their body condition recovery and the transition from late gestation to early lactation.

With respect to serum metabolite, in the prepartum period, the placenta transfers sugars such as GLU and fructose, lipids such as glycerol and free fatty acids (FFA), and free amino acids from the maternal to the fetus to supply energy, as well as fat and protein synthesis, by active transport and simple diffusion (27). In the postpartum period, mammary tissue requires large amounts of nutrients for the synthesis of milk components such as lactose, milk fat, and protein when lactation initiates after delivery (28). In the present study, GF yaks had significantly lower prepartum and postpartum serum GLU levels. It is speculated that due to the perinatal dam’s nutrition requirement and the deficient nutrient ingestion from conventional grazing, GF yaks could not have sufficient VFAs substrates from ruminal fermentation for gluconeogenesis. In contrast, this study’s significantly increased serum GLU levels in SF yaks enlightened us that perinatal nutritional supply provided more readily digestible carbohydrates for gluconeogenesis of transition yaks, an indispensable substrate of lactose synthesis in milk (29). Furthermore, yaks displayed significantly high serum glucose without nutrient output of lactation after early weaning. Serum levels of TG, NEFA, and BHBA can be the indicators revealing lipid and energy metabolism in animals (30, 31). NEFA in the blood reflects the degree of lipid mobilization, while BHBA indicates the process of incomplete lipid oxidation (32). Studies on dairy cows (11, 33) showed that under insufficient nutrient intake and NEB conditions, adipose tissue was mobilized during late pregnancy and early lactation due to fetal development and lactation. Our results show that yaks in the GF group experienced nutritional deficiency, as reflected in significantly higher NEFA and BHBA and lower body weight. As discussed above, lipids also play a crucial role in fetal energy supply and synthesis processes in the prepartum period and milk fat synthesis during lactation (27, 28). Under conventional grazing conditions, perinatal yaks could not generate enough lipid through ruminal fermentation and *de novo* lipogenesis (34). Therefore, the maternal adipose tissue was mobilized and utilized to alleviated the NEB and support the lipids requirement during transition period, leading to the increased lipolysis products NEFA and BHBA levels in GF yaks. With the nutritional supplementation treatment, SF and SW yaks’ nutrients intake increased, the NEB was thereby offset, favoring maternal pregnancy maintenance and perinatal transition. Supplementation and early weaning alleviated negative maternal energy balance and reduced lipid mobilization and ketone body when lactation was terminated. In the present study, the lipid transportation molecules APOB100 and VLDL of SF and SW yaks were significantly increased along with decreased lipid mobilization products NEFA and BHBA. The primary role of VLDL is to transport excess endogenous fatty acids (mostly TG) from the liver to provide energy to the tissues, which is crucial for lipid turnover and homeostasis (35). ApoB100, presents in every VLDL particle, is essential for VLDL structure and function (36). Elevated levels of serum APOB100 and VLDL indicate that nutritional supply and early weaning contributed to lipid transportation in periparturient yaks. In the postpartum period, we found that serum GLB and TP levels were significantly higher in SF and SW yaks compared to conventional grazing yaks, indicating the improvement of protein nutriture (37, 38) and hepatic function (39) of transition yaks with nutritional supplementation and early weaning treatment.

Regarding metabolic regulation hormones, it was demonstrated that improvement of nutritional supply contributed to decreased GC levels, reducing body lipid mobilization and promoting energy storage in the research on ewes (40). While poor nutritional status in yaks led to significantly reduced INS levels, and stimulation of body fat and glycogen breakdown (41). LEP, acting in the hypothalamus, regulates energy homeostasis and negatively correlates with serum NEFA and BHBA levels (42). IGF-I is structurally similar to INS (43) and performs a similar function in promoting the growth of animals. In our study, INS, LEP, and IGF-I levels were elevated in yaks who received nutrition supplementation during the perinatal transition. Increased INS levels are associated with nutritional and insulin resistance in late pregnancy and early lactation (44). Studies on dairy cows (45) have confirmed that cows at the end of gestation and early lactation experience a physiological state of high milk production and lipolysis, resulting in a physiological state of diminished biological effect of tissues on normal insulin doses (i.e., the onset of insulin resistance). Nutritional levels that may influence IGF-I secretion through insulin levels, fasting, malnutrition and food restriction have decreased plasma INS, IGF-I secretion, and IGF-I mRNA (46, 47). In contrast to the SF and SW yaks with nutrition supplementation, the GF yaks in our research with lower INS and IGF-I levels cohere with the previous studies. LEP levels were improved due to the enhanced maternal demand for milk fat synthesis (48). In transition yaks, an early weaning strategy elevated IGF-I levels. This result is consistent with the findings of early weaning on lactating Nellore cows (49), but the mechanism underlying the impact of early weaning on IGF-I secretion requires further study. With the increasing secretion of INS, LEP, and IGF-I involved in the regulation of nutritional metabolism, the anabolism of perinatal yaks with nutrition supplementation was potentially enhanced, and catabolism was downregulated following the results of body weight change.

Regarding reproduction, PROG positively correlates with litter size, milk yield, and body condition in dairy cows (50–52). Serum E_2_ also responds to the high fertility of the dam and coordinates the cycle of endometrial proliferation, differentiation, shedding, and regeneration (53–56). The significantly increased secretion of reproductive hormones E_2_ and PROG levels in SF and SW yaks imply that nutritional supplementation is beneficial in maintaining pregnancy and reproductive performance in periparturient yaks (57). Early weaning with a nutritional supplementation strategy further increased the serum FSH, LH, and PROG after weaning, which potentially promotes maturation and ovulation of the oocytes (58) and accelerates the next reproductive cycle, in accordance with the findings from a study on early weaning in beef cattle (59). The calving rate in the following year is considered a reliable indicator for evaluating the impact of perinatal nutrition supplementation and early weaning on the reproductive performance of yaks (60). The calving rates in the following year were 0% (GF), 16.7% (SF), and 83.3% (SW) in the present study. Adequate nutrition ingestion during the peripartum period was conducive to the recovery of the reproductive system, and an early weaning strategy can further expedite the reproductive cycle of yaks.

Metabolomic analysis identified differential metabolites associated with lipid metabolism, including lysophosphatidylcholine (LPC), phosphatidylcholine (PC), and branched fatty acid esters of hydroxy fatty acids (FAHFAs). PC is one of the major components of biofilms (61), and LPC is a metabolite produced by the enzymatic digestion of PC (62), which has a strong emulsifying capacity. Reportedly, LPC can enhance the uptake and utilization of glucose and effectively reduce blood glucose levels (61, 63, 64). Haetinger et al. (65), Papadopoulos et al. (66), and Boontiam et al. (67) have shown that LPC improves energy and nutrient utilization, especially lipid and protein digestibility, thus promotes production performance and gut health. However, studies on PC and LPC in ruminants are still scarce. Our experiment revealed a significant increase in LPC levels in SF yaks at −15 and 30 d. We speculated that the elevated serum levels of LPC are one of the reasons for elevated serum concentrations of INS. LPC can stimulate INS secretion through the protein kinase a (PKA)-related signaling pathway (68), thereby regulating glucose metabolism. In fat turnover metabolism, hepatic fat is transported in the form of PC, which is then transported by VLDL (69). Finally, PC generates glycerophosphatidylcholine (GPC) by activating phospholipase A2 and lysophosphatidylcholinesterase (70). At 90 d, SW yaks exhibited higher PC and LPC levels than SF yaks. This result implies that early weaning strategy facilitated the synthesis of PC and LPC, accelerated hepatic lipid turnover metabolism, reduced hepatic lipid accumulation, and thus improved the liver health in postpartum yaks. Our observation is consistent with previous findings showing significantly increased levels of ApoB100 in SW yaks compared to SF yaks at 90 d. In this study, serum of various FAHFA levels was notably reduced in SF yaks compared to GF yaks at −15, 30, and 90 d. FAHFAs constitute a novel class of biologically active lipid molecules discovered by Yore et al. (71) through lipidomic analysis. The FAHFAs family comprises a range of fatty acids (FA) and hydroxylated fatty acids (HFA). Within this family, FAHFA with identical FA and HFA compositions can generate ester bond position isomers resulting from variations in the positions of their ester bonds (71). Previous studies (72) have reported the presence of FAHFAs in both free and bound forms within the organism, with the initial discovery of bound FAHFAs occurring in TG. The reduction in FAHFA levels may correlate with the decrease in serum TG concentration after the treatment of SF. The impact of nutritional supplementation on perinatal serum FAHFA concentrations in our research is in accordance with the results obtained by Brezinova et al. (73), showing the negative correlation between nutritional levels and FAHFAs concentrations in maternal milk. Furthermore, FAHFAs [(18:2/18:3), (14:0/6:0)] in the SW group were significantly lower than those in the SF group at 90 d. With the previous observation that the lipid mobilization products NEFA and BHBA concentrations were also significantly reduced, it is inferred that early weaning had a contributory effect on the energy balance of the periparturient yaks. Consequently, the adipose mobilization of SW yaks was relieved, with decreasing levels of FAHFAs in the serum. The metabolism of milk protein synthesis rises once cows enter lactation, during which there is a significant increase in the demand for amino acids in the milk glands (74). During lactation, phenylalanine is a potentially limiting amino acid after methionine and lysine in milk (75). The serum metabolomics analysis also revealed, in comparison with GF yaks, the DL-m-tyrosine was significantly higher in SF yaks at −15 and 30 d, suggesting that periparturient nutritional supplementation may be able to improve the periparturient transition of yaks through the modulation of the protein turnover metabolism. Previous study reported that tyrosine has a function in relieving stress and fatigue (76). Yak dams with better nutritional supplementation in late pregnancy and early lactation had a higher level of tyrosine synthesis to reduce stress around parturition.

Our results exhibited three pathways related to lipid metabolism after KEGG pathways enrichment: biosynthesis of unsaturated fatty acids, serotonergic synapse, and regulation of lipolysis in adipocytes. At 30 d, the concentrations of significantly different metabolites, eicosapentaenoic, docosapentaenoic, and docosahexaenoic acids in the serum metabolome of SF yaks were significantly lower in the unsaturated fatty acid biosynthesis pathway as compared with those of GF yaks. This result enlightened us that yaks with nutritional supplementation had down-regulation of unsaturated fatty acid biosynthesis, which may contribute to more energy available for the demand of early lactating yaks and alleviate lipid mobilization. Serotonin (5-HT) is a neurotransmitter associated with feed intake and lipid metabolism (77, 78). It is reported that the down-regulation of 5-HT synthesis decreased lipogenesis, increased brown fat thermogenesis, and reduced body fat storage (79). In addition, hepatic gluconeogenesis could be promoted by 5-HT via 5-HT 2B receptors in hepatocytes (80). At 90 d, the 5-HT content in the serotonergic synapse pathway was reduced in the SF group compared with the GF group. The perinatal yaks benefited from nutrition supplementation through higher fat utilization, instead of lipogenesis, under the regulation of decreased 5-HT, in contrast, yaks responded to conventional grazing feeding with improved hepatic gluconeogenesis, which resulted from the greater 5-HT in order to maintain blood glucose level. TG in white adipose tissue (WAT) is the main energy reserve of the organism (81). Corticosterone regulates adipocyte lipolysis by acting on a glycoprotein-like phospholipase structural domain protein 2 (82). During energy deprivation, WAT undergoes a shift toward greater net rates of lipolysis, which can be defined as the hydrolysis of TG to generate fatty acids (FAs) and glycerol into the vasculature for utilization by other tissues as energy substrates (83). At 90 d postpartum, we observed decreased corticosterone, prostaglandin E2, and adenosine 5′-monophosphate concentrations in the regulation of lipolysis in adipocytes pathway in the SF group when compared with the GF group, corresponding to significantly lower NEFA and BHBA levels. Thus, nutrition supplementation for perinatal yaks reduced body fat mobilization by relieving NEB and down-regulating TG catabolic activity in regulating lipolysis in the adipocyte pathway (83). Overall, periparturient nutrient supplementation improved the nutritional status of yaks and consequent inhibition of lipid mobilization. The biosynthesis of unsaturated fatty acids and the regulation of lipolysis in adipocyte pathways involved in lipid metabolism were down-regulated by nutritional supply, which was also reflected in decreases of intermediate products of lipid metabolism, as well as the decreased lipolysis products NEFA and BHBA.

In the steroid hormone biosynthesis pathway, corticosterone tetrahydrocorticosterone levels were significantly reduced in SF yaks when compared to GF ones at −15 d. Based on the studies in poultry (84, 85), the decrease in corticosterone and tetrahydrocorticosterone concentrations likely indicates that periparturient stress is alleviated in yaks under nutritional supplementation. Meanwhile, the differential metabolites of androstenedione, dehydroepiandrosterone, and epitestosterone concentrations in serum hormones and hormone derivatives significantly increased in the SF group compared with the GF group at −15, 30, and 90 d. The better nutritional status of the yak dam could participate in the regulation of the immune, metabolism, and reproduction systems by stimulating the secretion of androsterone and dehydroepiandrosterone (86), reducing the deposition of lipids through the enhancement of hepatic lipid oxidation and inhibition of lipid synthesis in the liver (87, 88). Furthermore, we identified two reproductive-related metabolic pathways at −15 d, the prolactin signaling pathway and ovarian steroidogenesis, using KEGG pathway enrichment analysis. The ovarian steroidogenic pathway involves the transportation of cholesterol (CHO) to the inner mitochondrial membrane, where it is converted to pregnenolone. Pregnenolone is then transformed into progesterone, followed by conversion to 17α-hydroxy pregnenolone. This compound is further metabolized into dehydroepiandrosterone and then into androstenedione. Finally, androstenedione is converted to E_2_ (89). PROG is involved in the prolactin signaling pathway, generating androstenedione. This conversion occurs through the action of 17α-monooxygenase and 17α-hydroxyprogesterone deacetylase. Subsequently, androstenedione is metabolized via the estrogen signaling pathway to produce E_2_ (90, 91). SF yaks exhibited lower pregnenolone, androstenedione, estrone, and PROG concentrations in the ovarian steroidogenesis pathway, as well as significantly reduced androstenedione, estrone, and progesterone levels in the prolactin signaling pathway compared to GF yaks. Interestingly, serum biochemistry showed that the serum CHO, PROG and E2 levels increased with nutritional supplementation during the perinatal period. We speculated that improved nutritional intake in SF yaks may contribute to better maintenance of pregnancy and higher reproductive hormone levels, thus the demands of PROG and E_2_ reduced, resulting in down-regulation of the above pathways and decreased concentrations of intermediary metabolites. From the point of view of GF yaks, serum levels of TP, TG, and the reproductive hormones PROG and E_2_ were significantly lower, and the fat mobilization products NEFA and BHBA were significantly increased. Serum Biochemistry, reproductive hormones, and metabolomics results provided the evidence that yaks in late gestation maintain pregnancy and ensure fetal development (57), probably through up-regulating the biological process of E_2_ and PROG production in the prolactin signaling pathway and ovarian steroidogenesis pathway and lipid mobilization for energy supply (89) when perinatal nutrition and pregnant status are poor.

## 5 Conclusion

These results demonstrated that yaks in conventional grazing system had poor recovery and adaptation to transition period because of nutrition deficiency. Perinatal nutrition supplementation contributed to body condition recovery and improved glucose and nitrogen metabolism of yaks under the regulation of increased INS, LEP, and IGF-I secretion. The lipid metabolism of periparturient yaks benefited from the nutrition supplementation, which also promoted the maintenance of pregnancy and the recovery of the reproductive system after parturition by stimulating the secretion of the reproductive hormones E_2_ and PROG. The early weaning strategy (60 d) combined with nutritional supplementation further improved postpartum recovery and lipid metabolism adaptation to the transition period. It subsequently accelerated the reproductive cycle by enhancing the reproductive hormones FSH and LH secretion of transition yaks.

## Data Availability

The data presented in the study are deposited in Figshare database, DOI: 10.6084/m9.figshare.27894057. https://figshare.com/s/143f6db2b15d789daea9.

## Footnotes

^1^ https://www.genome.jp/kegg/pathway.html

^2^ https://hmdb.ca/metabolites

^3^ http://www.lipidmaps.org/
