# Supplementary material for: Effects of perinatal nutrition supplementation and early weaning on serum biochemistry, metabolomics, and reproduction in yaks
Source: Front Vet Sci. 2024 Dec 19;11:1443856. doi: 10.3389/fvets.2024.1443856 (PMC11694451; doi:10.3389/fvets.2024.1443856)
Supplement: Supplementary file 1 [file Table_1.DOCX]

Supplementary Material

**Table S1. Nutrient levels of milk replacer and starter concentrate (DM basis).**

| Nutrient Level^1^ | Milk replacer | Starter concentrate |
| --- | --- | --- |
| CP | 24.85 | 24.53 |
| EE | 12.11 | 8.68 |
| NDF | - | - |
| ADF | - | - |
| Ash | 6.11 | 6.65 |
| Ca | 1.00 | 1.48 |
| P | 0.69 | 0.83 |

^1^CP=crude protein, EE=ether extract, Ca=calcium, P=phosphorus.

**Table S2. Screening results of significantly different metabolites of yak serum in the perinatal period.**

| Compared samples^1^ | Num of Total Ident. | Num of Total Sig. | Num of Sig, up | Num of Sig, down |
| --- | --- | --- | --- | --- |
| SF-15d.vs.GF-15d_pos | 848 | 224 | 72 | 152 |
| SF30d.vs.GF30d_pos | 848 | 275 | 125 | 150 |
| SF90d.vs.GF90d_pos | 848 | 254 | 124 | 130 |
| SW90d.vs.GF90d_pos | 848 | 246 | 116 | 130 |
| SW90d.vs. SF90d_pos | 848 | 77 | 26 | 51 |
| SF-15d.vs.GF-15d_neg | 350 | 118 | 29 | 89 |
| SF30d.vs.GF30d_neg | 350 | 120 | 52 | 68 |
| SF90d.vs.GF90d_neg | 350 | 108 | 54 | 54 |
| SW90d.vs.GF90d_neg | 350 | 118 | 53 | 65 |
| SW90d.vs. SF90d_neg | 350 | 43 | 23 | 20 |

^1^Yaks in the GF, SF and SW groups were free grazing on the same pasture from −30 to 90 day relative to parturition, being released to pasture at 08:00 and returning to barn at 18:00. Yaks in SF and SW groups received total mixed ration supplementation in barn during the night (18:00-08:00) from −30 to 90 day. Calves in the SW group were early weaned and separated from the dam at 60 day postpartum. pos=positive mode, neg=negative mode.


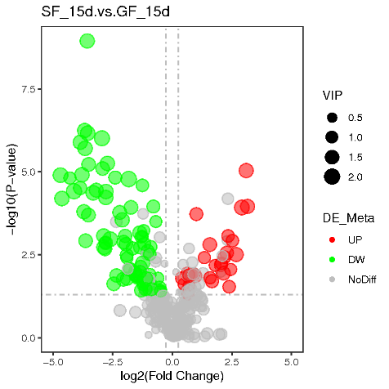

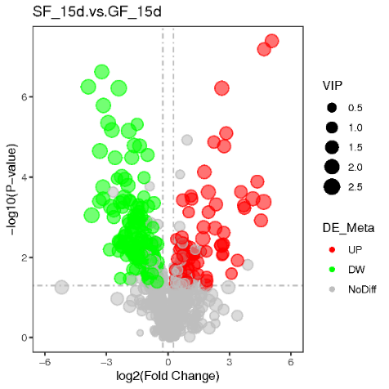

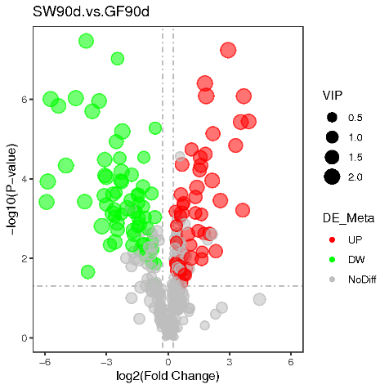

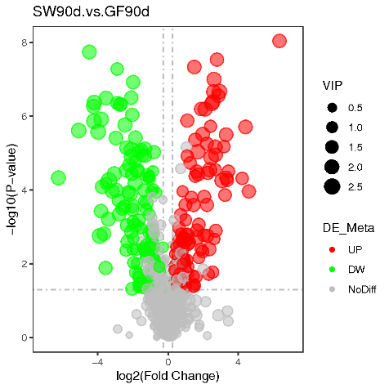

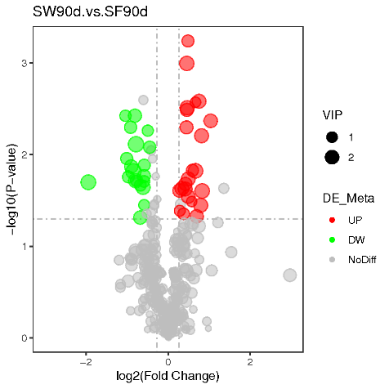

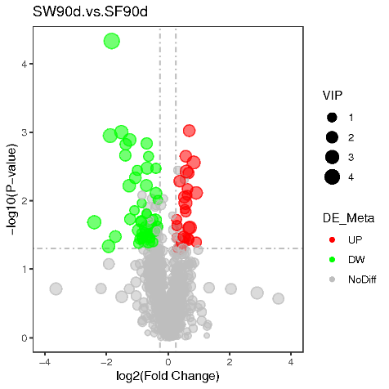

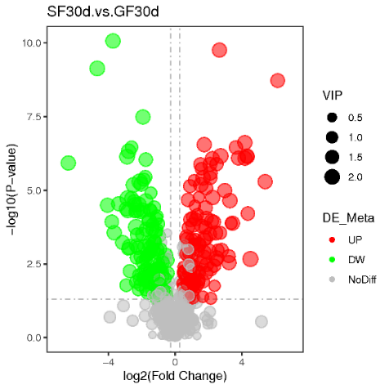

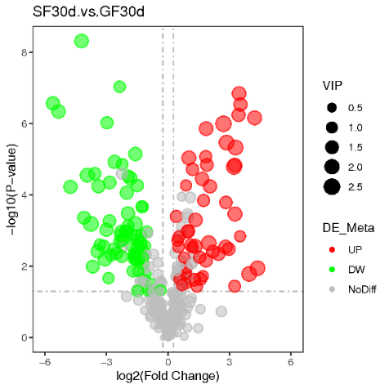

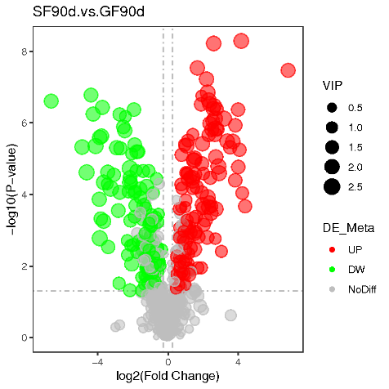

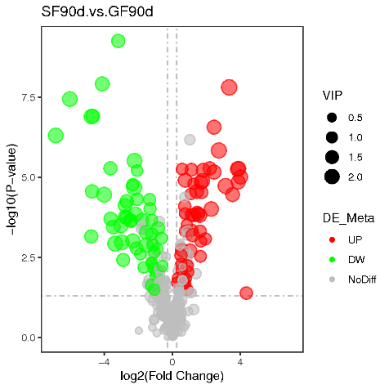
**Figure S1.** Volcano map of clustering of the effects of perinatal nutritional supplementation and early weaning on serum metabolites.

(B)

(A)

(J)

(I)

(H)

(G)

(F)

(E)

(C)

(D)

Note: Yaks in the GF, SF and SW groups were free grazing on the same pasture from −30 to 90 day relative to parturition, being released to pasture at 08:00 and returning to barn at 18:00. Yaks in SF and SW groups received total mixed ration supplementation in barn during the night (18:00-08:00) from −30 to 90 day. Calves in the SW group were early weaned and separated from the dam at 60 day postpartum. (A) (B) SF vs. GF in positive and negative modes at -15d, (C)(D) SF vs.GF in positive and negative modes at 30d, (E) (F) SF vs. GF in positive and negative modes at 90d. (G) (H) SW vs. GF in positive and negative modes at 90d. (I) (J) SW vs. SF in positive and negative modes at 90d.

**Table S3. Enriched pathways and significantly different metabolites in positive mode in the comparison of SF vs. GF at 15 days before parturition**

| No. | Metabolic pathways | Significantly different metabolites | P-value |
| --- | --- | --- | --- |
| 1 | Steroid hormone biosynthesis＊ | Pregnenolone↓; Etiocholanolone↑; Cortodoxone↓; Corticosterone↓; Androsterone↑; Androstenedione↓; Tetrahydrocorticosterone↓; Estrone↓; Progesterone↓ | 0.001 |
| 2 | Prolactin signaling pathway＊ | Androstenedione↓; Estrone↓; Progesterone↓ | 0.010 |
| 3 | Aldosterone synthesis and secretion＊ | Pregnenolone↓; Corticosterone↓; Progesterone↓ | 0.010 |
| 4 | Ovarian steroidogenesis＊ | Pregnenolone↓; Androstenedione↓; Estrone↓; Progesterone↓ | 0.022 |
| 5 | Cortisol synthesis and secretion | Pregnenolone↓; Cortodoxone↓; Progesterone↓ | 0.073 |
| 6 | Cushing's syndrome | Pregnenolone↓; Cortodoxone↓; Progesterone↓ | 0.073 |
| 7 | Biosynthesis of unsaturated fatty acids | Eicosapentaenoic acid↓; Docosapentaenoic acid↓ | 0.125 |
| 8 | Fatty acid degradation | L-Palmitoylcarnitine↓ | 0.224 |
| 9 | Amino sugar and nucleotide sugar metabolism | N-Acetylneuraminic acid↓ | 0.224 |
| 10 | One carbon pool by folate | Folinic acid↑ | 0.224 |
| 11 | Fatty acid metabolism | L-Palmitoylcarnitine↓ | 0.224 |
| 12 | Endocrine resistance | Androstenedione↓ | 0.224 |
| 13 | Oocyte meiosis | Progesterone↓ | 0.224 |
| 14 | Progesterone-mediated oocyte maturation | Progesterone↓ | 0.224 |
| 15 | Prion diseases | Corticosterone↓ | 0.224 |
| 16 | Breast cancer | Progesterone↓ | 0.224 |
| 17 | Prostate cancer | Androstenedione↓; Progesterone↓ | 0.312 |
| 18 | Pantothenate and CoA biosynthesis | Pantothenic acid↑ | 0.400 |
| 19 | Arginine biosynthesis | N-Acetylornithine↓ | 0.537 |
| 20 | Lysine degradation | N6,N6,N6-Trimethyl-L-lysine↓ | 0.537 |
| 21 | alpha-Linolenic acid metabolism | 13(S)-HOTrE↓ | 0.537 |
| 22 | Regulation of lipolysis in adipocytes | Corticosterone↓ | 0.537 |
| 23 | Pathways in cancer | Androstenedione↓; Progesterone↓ | 0.614 |
| 24 | Vitamin B6 metabolism | Pyridoxine↓ | 1.000 |
| 25 | Porphyrin and chlorophyll metabolism | Bilirubin↓ | 1.000 |

Note: ↑ and ↓ in the Significantly different metabolites signify that the metabolite is significantly up-regulated or down-regulated in the metabolic pathway, and * denotes KEGG pathways that are significantly enriched in the differential metabolite, same as the below.

**Table S4. Enriched pathways and significantly different metabolites in negative mode in the comparison of SF vs. GF at 15 days before parturition**

| No. | Metabolic pathways | Significantly different metabolites | P-value |
| --- | --- | --- | --- |
| 1 | Dopaminergic synapse | Levodopa↑; Homovanillic acid↓ | 0.159 |
| 2 | Ovarian steroidogenesis | Arachidonic acid↓; 12(S)-HETE↓ | 0.159 |
| 3 | Regulation of lipolysis in adipocytes | L-Adrenaline↑; Arachidonic acid↓ | 0.159 |
| 4 | Aldosterone synthesis and secretion | Arachidonic acid↓; 12(S)-HETE↓ | 0.159 |
| 5 | Bile secretion | Cholic acid↓; Deoxycholic Acid↓; Thromboxane B2↓ | 0.294 |
| 6 | Tyrosine metabolism | Levodopa↑; L-Adrenaline↑; Homovanillic acid↓ | 0.385 |
| 7 | Fatty acid elongation | Palmitic acid↓ | 0.403 |
| 8 | Fatty acid degradation | Palmitic acid↓ | 0.403 |
| 9 | Valine, leucine and isoleucine biosynthesis | 2-Isopropylmalic acid↓ | 0.403 |
| 10 | Fatty acid metabolism | Palmitic acid↓ | 0.403 |
| 11 | cAMP signaling pathway | L-Adrenaline↑ | 0.403 |
| 12 | Longevity regulating pathway | Resveratrol↓ | 0.403 |
| 13 | Ferroptosis | Arachidonic acid↓ | 0.403 |
| 14 | Necroptosis | Arachidonic acid↓ | 0.403 |
| 15 | Adrenergic signaling in cardiomyocytes | L-Adrenaline↑ | 0.403 |
| 16 | Vascular smooth muscle contraction | Arachidonic acid↓ | 0.403 |
| 17 | Fc gamma R-mediated phagocytosis | Arachidonic acid↓ | 0.403 |
| 18 | Long-term depression | Arachidonic acid↓ | 0.403 |
| 19 | Inflammatory mediator regulation of TRP channels | Arachidonic acid↓ | 0.403 |
| 20 | GnRH signaling pathway | Arachidonic acid↓ | 0.403 |
| 21 | Prolactin signaling pathway | Levodopa↑ | 0.403 |
| 22 | Renin secretion | L-Adrenaline↑ | 0.403 |
| 23 | Parkinson's disease | Levodopa↑ | 0.403 |
| 24 | Cocaine addiction | Levodopa↑ | 0.403 |
| 25 | Amphetamine addiction | Levodopa↑ | 0.403 |
| 2+ | Alcoholism | Levodopa↑ | 0.403 |
| 27 | Biosynthesis of unsaturated fatty acids | Arachidic Acid↓; Stearic acid↓; Nervonic acid↓; Palmitic acid↓; Arachidonic acid↓ | 0.465 |
| 28 | Fatty acid biosynthesis | Stearic acid; Palmitic acid↓; Palmitoleic Acid↓ | 0.678 |
| 29 | Primary bile acid biosynthesis | Cholic acid↓ | 1.000 |
| 30 | Linoleic acid metabolism | Arachidonic acid↓ | 1.000 |
| 31 | Pyruvate metabolism | 2-Isopropylmalic acid↓ | 1.000 |
| 32 | Fc epsilon RI signaling pathway | Arachidonic acid↓ | 1.000 |
| 33 | Retrograde endocannabinoid signaling | Arachidonic acid↓ | 1.000 |
| 34 | Oxytocin signaling pathway | Arachidonic acid↓ | 1.000 |
| 35 | Pyrimidine metabolism | Pseudouridine↓; 2-Deoxyuridine↓ | 1.000 |
| 36 | Arachidonic acid metabolism | Thromboxane B2↓; Arachidonic acid; 12(S)-HETE↓ | 1.000 |

**Table S5. Enriched pathways and significantly different metabolites in positive mode in the comparison of SF vs. GF at 30 days after parturition**

| No. | Metabolic pathways | Significantly different metabolites | P-value |
| --- | --- | --- | --- |
| 1 | Biosynthesis of unsaturated fatty acids＊ | Eicosapentaenoic acid↓; Docosapentaenoic acid↓; Docosahexaenoic acid↓ | 0.025 |
| 2 | Glutathione metabolism | L-Glutamate↑; L-Pyroglutamic acid↓ | 0.087 |
| 3 | Renin secretion | Prostaglandin E2↓; Adenosine 5'-monophosphate↓ | 0.087 |
| 4 | alpha-Linolenic acid metabolism | 13(S)-HOTrE↓; Traumatic acid↑ | 0.212 |
| 5 | Prolactin signaling pathway | Androstenedione↓; Estrone↓ | 0.212 |
| 6 | Regulation of lipolysis in adipocytes | Prostaglandin E2↓; Adenosine 5'-monophosphate↓ | 0.212 |
| 7 | Steroid hormone biosynthesis | Cortisone↓; 7α-Hydroxytestosterone↑; Etiocholanolone↑; Androsterone↑; Androstenedione↓; Tetrahydrocorticosterone↓; Estrone↓ | 0.238 |
| 8 | Phosphonate and phosphinate metabolism | Bialaphos↓ | 0.299 |
| 9 | Neomycin, kanamycin and gentamicin biosynthesis | L-Glutamate↑ | 0.299 |
| 10 | Linoleic acid metabolism | 13-OxoODE↑ | 0.299 |
| 11 | One carbon pool by folate | Folinic acid↑ | 0.299 |
| 12 | Riboflavin metabolism | Vitamin B2↑ | 0.299 |
| 13 | Nitrogen metabolism | L-Glutamate↑ | 0.299 |
| 14 | Endocrine resistance | Androstenedione↓ | 0.299 |
| 15 | cGMP-PKG signaling pathway | Adenosine 5'-monophosphate↓ | 0.299 |
| 16 | FoxO signaling pathway | Adenosine 5'-monophosphate↓ | 0.299 |
| 17 | mTOR signaling pathway | Adenosine 5'-monophosphate↓ | 0.299 |
| 18 | PI3K-Akt signaling pathway | Adenosine 5'-monophosphate↓ | 0.299 |
| 19 | AMPK signaling pathway | Adenosine 5'-monophosphate↓ | 0.299 |
| 20 | Longevity regulating pathway | Adenosine 5'-monophosphate↓ | 0.299 |
| 21 | Cholinergic synapse | Choline↓ | 0.299 |
| 22 | Olfactory transduction | Adenosine 5'-monophosphate↓ | 0.299 |
| 23 | Oxytocin signaling pathway | Prostaglandin E2↓ | 0.299 |
| 24 | Leishmaniasis | Prostaglandin E2↓ | 0.299 |
| 25 | Amoebiasis | Prostaglandin E2↓ | 0.299 |
| 2+ | Human papillomavirus infection | Prostaglandin E2↓ | 0.299 |
| 27 | Rheumatoid arthritis | Prostaglandin E2↓ | 0.299 |
| 28 | Pathways in cancer | Cortisone↓; Prostaglandin E2↓; Androstenedione↓ | 0.361 |
| 29 | Taurine and hypotaurine metabolism | L-Glutamate↑ | 0.511 |
| 30 | D-Glutamine and D-glutamate metabolism | L-Glutamate↑ | 0.511 |
| 31 | Glycerophospholipid metabolism | Choline↓ | 0.511 |
| 32 | Antifolate resistance | Adenosine 5'-monophosphate↓ | 0.511 |
| 33 | Inflammatory mediator regulation of TRP channels | Prostaglandin E2↓ | 0.511 |
| 34 | Aldosterone-regulated sodium reabsorption | Cortisone↓ | 0.511 |
| 35 | African trypanosomiasis | Prostaglandin E2↓ | 0.511 |
| 36 | Choline metabolism in cancer | Choline↓ | 0.511 |
| 37 | cAMP signaling pathway | Prostaglandin E2↓; Adenosine 5'-monophosphate↓ | 0.581 |
| 38 | Prostate cancer | Cortisone; Androstenedione↓ | 0.634 |
| 39 | Arginine biosynthesis | L-Glutamate↑ | 1.000 |
| 40 | Glycine, serine and threonine metabolism | Choline↓ | 1.000 |
| 41 | Glyoxylate and dicarboxylate metabolism | L-Glutamate↑ | 1.000 |
| 42 | Butanoate metabolism | L-Glutamate↑ | 1.000 |
| 43 | Serotonergic synapse | Prostaglandin E2↓ | 1.000 |
| 44 | Ovarian steroidogenesis | Androstenedione↓; Estrone↓ | 1.000 |
| 45 | Bile secretion | Prostaglandin E2↓; Fexofenadine↓; Choline↓ | 1.000 |

**Table S6. Enriched pathways and significantly different metabolites in negative mode in the comparison of SF vs. GF at 30 days after parturition**

| No. | Metabolic pathways | Significantly different metabolites | P-value |
| --- | --- | --- | --- |
| 1 | Fatty acid biosynthesis | Stearic acid↓; Palmitic acid↓; Myristic Acid↓; Palmitoleic Acid↓ | 0.054 |
| 2 | Vitamin digestion and absorption | Menaquinone↑; Riboflavin-5-phosphate↑ | 0.081 |
| 3 | Ubiquinone and other terpenoid-quinone biosynthesis | Menaquinone↑; 4-Hydroxybenzoic acid↑ | 0.200 |
| 4 | Fatty acid elongation | Palmitic acid↓ | 0.290 |
| 5 | Fatty acid degradation | Palmitic acid↓ | 0.290 |
| 6 | Arginine biosynthesis | Citrulline↓ | 0.290 |
| 7 | Cysteine and methionine metabolism | S-Sulfo-L-cysteine | 0.290 |
| 8 | Riboflavin metabolism | Riboflavin-5-phosphate↑ | 0.290 |
| 9 | Folate biosynthesis | 4-Hydroxybenzoic acid↑ | 0.290 |
| 10 | Fatty acid metabolism | Palmitic acid↓ | 0.290 |
| 11 | cAMP signaling pathway | L-Adrenaline↑ | 0.290 |
| 12 | Longevity regulating pathway | Resveratrol↓ | 0.290 |
| 13 | Adrenergic signaling in cardiomyocytes | L-Adrenaline↑ | 0.290 |
| 14 | Renin secretion | L-Adrenaline↑ | 0.290 |
| 15 | Biosynthesis of unsaturated fatty acids | Arachidic Acid↓; Stearic acid↓; Palmitic acid↓; Erucic acid↓ | 0.427 |
| 16 | Primary bile acid biosynthesis | Cholic acid↓ | 0.500 |
| 17 | Oxidative phosphorylation | Riboflavin-5-phosphate↑ | 0.500 |
| 18 | Ovarian steroidogenesis | 12(S)-HETE↓ | 0.500 |
| 19 | Regulation of lipolysis in adipocytes | L-Adrenaline↑ | 0.500 |
| 20 | Aldosterone synthesis and secretion | 12(S)-HETE↓ | 0.500 |
| 21 | Phenylalanine metabolism | 4-Hydroxybenzoic acid↑ | 1.000 |
| 22 | Neuroactive ligand-receptor interaction | L-Adrenaline↑ | 1.000 |

**Table S7. Enriched pathways and significantly different metabolites in positive mode in the comparison of SF vs. GF at 90 days after parturition**

| No. | Metabolic pathways | Significantly different metabolites | P-value |
| --- | --- | --- | --- |
| 1 | Serotonergic synapse＊ | Serotonin↓; Prostaglandin E2↓; Prostaglandin J2↓ | 0.023 |
| 2 | Regulation of lipolysis in adipocytes＊ | Corticosterone↓; Prostaglandin E2↓; Adenosine 5'-monophosphate↓ | 0.023 |
| 3 | Tyrosine metabolism | Hydroquinone↓; 3,4-Dihydroxyphenylpropionic acid↓; 3-Methoxytyramine↓ | 0.072 |
| 4 | cAMP signaling pathway | Serotonin↓; Prostaglandin E2↓; Adenosine 5'-monophosphate↓ | 0.072 |
| 5 | Citrate cycle (TCA cycle) | alpha-Ketoglutaric acid↑; cis-Aconitic acid↑ | 0.082 |
| 6 | Inflammatory mediator regulation of TRP channels | Serotonin↓; Prostaglandin E2↓ | 0.082 |
| 7 | Renin secretion | Prostaglandin E2↓; Adenosine 5'-monophosphate↓ | 0.082 |
| 8 | Bile secretion | Glycocholic acid↓; Serotonin↓; Prostaglandin E2↓; Fexofenadine↓; Bilirubin↓ | 0.117 |
| 9 | alpha-Linolenic acid metabolism | 13(S)-HOTrE↓; Traumatic acid↑ | 0.201 |
| 10 | Glyoxylate and dicarboxylate metabolism | alpha-Ketoglutaric acid↑; cis-Aconitic acid↑ | 0.201 |
| 11 | Pentose and glucuronate interconversions | alpha-Ketoglutaric acid↑ | 0.290 |
| 12 | Ascorbate and aldarate metabolism | alpha-Ketoglutaric acid↑ | 0.290 |
| 13 | cGMP-PKG signaling pathway | Adenosine 5'-monophosphate↓ | 0.290 |
| 14 | FoxO signaling pathway | Adenosine 5'-monophosphate↓ | 0.290 |
| 15 | mTOR signaling pathway | Adenosine 5'-monophosphate↓ | 0.290 |
| 16 | PI3K-Akt signaling pathway | Adenosine 5'-monophosphate↓ | 0.290 |
| 17 | AMPK signaling pathway | Adenosine 5'-monophosphate↓ | 0.290 |
| 18 | Longevity regulating pathway | Adenosine 5'-monophosphate↓ | 0.290 |
| 19 | Ferroptosis | L-Cystine↓ | 0.290 |
| 20 | Gap junction | Serotonin↓ | 0.290 |
| 21 | Dopaminergic synapse | 3-Methoxytyramine↓ | 0.290 |
| 22 | Olfactory transduction | Adenosine 5'-monophosphate↓ | 0.290 |
| 23 | Oxytocin signaling pathway | Prostaglandin E2↓ | 0.290 |
| 24 | Prion diseases | Corticosterone↓ | 0.290 |
| 25 | Leishmaniasis | Prostaglandin E2↓ | 0.290 |
| 2+ | Amoebiasis | Prostaglandin E2↓ | 0.290 |
| 27 | Human papillomavirus infection | Prostaglandin E2↓ | 0.290 |
| 28 | Rheumatoid arthritis | Prostaglandin E2↓ | 0.290 |
| 29 | Primary bile acid biosynthesis | Glycocholic acid↓ | 0.497 |
| 30 | Cysteine and methionine metabolism | L-Cystine↓ | 0.497 |
| 31 | Taurine and hypotaurine metabolism | alpha-Ketoglutaric acid↑ | 0.497 |
| 32 | D-Glutamine and D-glutamate metabolism | alpha-Ketoglutaric acid↑ | 0.497 |
| 33 | Antifolate resistance | Adenosine 5'-monophosphate↓ | 0.497 |
| 34 | Synaptic vesicle cycle | Serotonin↓ | 0.497 |
| 35 | Cholesterol metabolism | Glycocholic acid↓ | 0.497 |
| 36 | African trypanosomiasis | Prostaglandin E2↓ | 0.497 |
| 37 | Arachidonic acid metabolism | Prostaglandin E2↓; Prostaglandin J2↓ | 0.578 |
| 38 | Neuroactive ligand-receptor interaction | Serotonin↓; Prostaglandin E2↓ | 0.578 |
| 39 | Taste transduction | Serotonin↓; Adenosine 5'-monophosphate↓ | 0.578 |
| 40 | Histidine metabolism | alpha-Ketoglutaric acid↑; Carnosine↑ | 0.626 |
| 41 | Purine metabolism | Xanthine↑; Adenosine 5'-monophosphate↓; Hypoxanthine | 0.716 |
| 42 | Steroid hormone biosynthesis | 7α-Hydroxytestosterone↑; Corticosterone↓; Androsterone↑; Tetrahydrocorticosterone↓; Estrone↓ | 0.775 |
| 43 | Arginine biosynthesis | alpha-Ketoglutaric acid↑ | 1.000 |
| 44 | Butanoate metabolism | alpha-Ketoglutaric acid↑ | 1.000 |
| 45 | Biosynthesis of unsaturated fatty acids | Eicosapentaenoic acid↓ | 1.000 |
| 46 | Prolactin signaling pathway | Estrone↓ | 1.000 |
| 47 | Aldosterone synthesis and secretion | Corticosterone↓ | 1.000 |
| 48 | Tryptophan metabolism | Serotonin↓; Indole-3-acetic acid↑ | 1.000 |

**Table S8. Enriched pathways and significantly different metabolites in negative mode in the comparison of SF vs. GF at 90 days after parturition**

| No. | Metabolic pathways | Significantly different metabolites | P-value |
| --- | --- | --- | --- |
| 1 | Vitamin digestion and absorption | Menaquinone↑; Riboflavin-5-phosphate↑ | 0.081 |
| 2 | Arachidonic acid metabolism | Prostaglandin G2↓; Lipoxin B4↓; Prostaglandin D2↑; 12(S)-HETE↓ | 0.179 |
| 3 | Biosynthesis of amino acids | Tryptophan↑; Citric acid↑; S-Sulfo-L-cysteine↑; Citrulline↑ | 0.179 |
| 4 | African trypanosomiasis | Tryptophan↑; Prostaglandin D2↑ | 0.200 |
| 5 | Galactose metabolism | Inositol↑ | 0.290 |
| 6 | Ascorbate and aldarate metabolism | Inositol↑ | 0.290 |
| 7 | Arginine biosynthesis | Citrulline↑ | 0.290 |
| 8 | Caffeine metabolism | Theophylline↑ | 0.290 |
| 9 | Cysteine and methionine metabolism | S-Sulfo-L-cysteine↑ | 0.290 |
| 10 | Inositol phosphate metabolism | Inositol↑ | 0.290 |
| 11 | Riboflavin metabolism | Riboflavin-5-phosphate↑ | 0.290 |
| 12 | Carbon metabolism | Citric acid↑ | 0.290 |
| 13 | Phosphatidylinositol signaling system | Inositol↑ | 0.290 |
| 14 | Longevity regulating pathway | Resveratrol↓ | 0.290 |
| 15 | Asthma | Prostaglandin D2↑ | 0.290 |
| 16 | Serotonergic synapse | Prostaglandin G2↓; Tryptophan↑; Prostaglandin D2↑ | 0.404 |
| 17 | Biosynthesis of unsaturated fatty acids | Arachidic Acid↓; Stearic acid↓; Lignoceric Acid↓; Nervonic acid↓ | 0.427 |
| 18 | Citrate cycle (TCA cycle) | Citric acid↑ | 0.500 |
| 19 | Oxidative phosphorylation | Riboflavin-5-phosphate↑ | 0.500 |
| 20 | Glycine, serine and threonine metabolism | Tryptophan↑ | 0.500 |
| 21 | Glyoxylate and dicarboxylate metabolism | Citric acid↑ | 0.500 |
| 22 | ABC transporters | Inositol↑ | 0.500 |
| 23 | Fc epsilon RI signaling pathway | Prostaglandin D2↑ | 0.500 |
| 24 | Ovarian steroidogenesis | 12(S)-HETE↓ | 0.500 |
| 25 | Aldosterone synthesis and secretion | 12(S)-HETE↓ | 0.500 |
| 26 | Mineral absorption | Tryptophan↑ | 0.500 |
| 27 | 2-Oxocarboxylic acid metabolism | Tryptophan↑; Citric acid↑ | 0.573 |
| 28 | Ubiquinone and other terpenoid-quinone biosynthesis | Menaquinone↑ | 1.000 |
| 29 | Alanine, aspartate and glutamate metabolism | Citric acid↑ | 1.000 |
| 30 | Aminoacyl-tRNA biosynthesis | Tryptophan↑ | 1.000 |
| 31 | Neuroactive ligand-receptor interaction | Prostaglandin D2↑ | 1.000 |
| 32 | Platelet activation | Prostaglandin G2↓ | 1.000 |
| 33 | Protein digestion and absorption | Tryptophan↑ | 1.000 |

**Table S9. Enriched pathways and significantly different metabolites in positive mode in the comparison of SW vs. GF at 90 days after parturition**

| No. | Metabolic pathways | Significantly different metabolites | P-value |
| --- | --- | --- | --- |
| 1 | Serotonergic synapse＊ | Serotonin↓; Prostaglandin E2↓; Prostaglandin J2↓ | 0.018 |
| 2 | Bile secretion | Glycocholic acid↓; Serotonin↓; Prostaglandin E2↓; Fexofenadine↓; Bilirubin↓ | 0.059 |
| 3 | Tyrosine metabolism | Hydroquinone↓; 3,4-Dihydroxyphenylpropionic acid↓; 3-Methoxytyramine↓ | 0.060 |
| 4 | cAMP signaling pathway | Serotonin↓; Prostaglandin E2↓; Adenosine 5'-monophosphate↓ | 0.060 |
| 5 | Antifolate resistance | Methionine↓; Adenosine 5'-monophosphate↓ | 0.072 |
| 6 | Inflammatory mediator regulation of TRP channels | Serotonin↓; Prostaglandin E2↓ | 0.072 |
| 7 | Renin secretion | Prostaglandin E2↓; Adenosine 5'-monophosphate↓ | 0.072 |
| 8 | alpha-Linolenic acid metabolism | 13(S)-HOTrE↓; Traumatic acid↑ | 0.178 |
| 9 | Regulation of lipolysis in adipocytes | Prostaglandin E2↓; Adenosine 5'-monophosphate↓ | 0.178 |
| 10 | Pentose and glucuronate interconversions | alpha-Ketoglutaric acid↑ | 0.271 |
| 11 | Ascorbate and aldarate metabolism | alpha-Ketoglutaric acid↑ | 0.271 |
| 12 | Metabolism of xenobiotics by cytochrome P450 | 2-Naphthol↑ | 0.271 |
| 13 | cGMP-PKG signaling pathway | Adenosine 5'-monophosphate↓ | 0.271 |
| 14 | FoxO signaling pathway | Adenosine 5'-monophosphate↓ | 0.271 |
| 15 | mTOR signaling pathway | Adenosine 5'-monophosphate↓ | 0.271 |
| 16 | PI3K-Akt signaling pathway | Adenosine 5'-monophosphate↓ | 0.271 |
| 17 | AMPK signaling pathway | Adenosine 5'-monophosphate↓ | 0.271 |
| 18 | Longevity regulating pathway | Adenosine 5'-monophosphate↓ | 0.271 |
| 19 | Gap junction | Serotonin↓ | 0.271 |
| 20 | Dopaminergic synapse | 3-Methoxytyramine↓ | 0.271 |
| 21 | Olfactory transduction | Adenosine 5'-monophosphate↓ | 0.271 |
| 22 | Oxytocin signaling pathway | Prostaglandin E2↓ | 0.271 |
| 23 | Leishmaniasis | Prostaglandin E2↓ | 0.271 |
| 24 | Amoebiasis | Prostaglandin E2↓ | 0.271 |
| 25 | Human papillomavirus infection | Prostaglandin E2↓ | 0.271 |
| 2+ | Rheumatoid arthritis | Prostaglandin E2↓ | 0.271 |
| 27 | Arachidonic acid metabolism | Prostaglandin E2↓; Prostaglandin J2↓ | 0.296 |
| 28 | Neuroactive ligand-receptor interaction | Serotonin↓; Prostaglandin E2↓ | 0.296 |
| 29 | Taste transduction | Serotonin↓; Adenosine 5'-monophosphate↓ | 0.296 |
| 30 | Tryptophan metabolism | Kynurenic acid↓; Serotonin↓; Indole-3-acetic acid↑ | 0.341 |
| 31 | Citrate cycle (TCA cycle) | alpha-Ketoglutaric acid↑ | 0.470 |
| 32 | Primary bile acid biosynthesis | Glycocholic acid↓ | 0.470 |
| 33 | Cysteine and methionine metabolism | Methionine↓ | 0.470 |
| 34 | Taurine and hypotaurine metabolism | alpha-Ketoglutaric acid↑ | 0.470 |
| 35 | D-Glutamine and D-glutamate metabolism | alpha-Ketoglutaric acid↑ | 0.470 |
| 36 | Synaptic vesicle cycle | Serotonin↓ | 0.470 |
| 37 | Cholesterol metabolism | Glycocholic acid↓ | 0.470 |
| 38 | African trypanosomiasis | Prostaglandin E2↓ | 0.470 |
| 39 | Histidine metabolism | 1-Methylhistidine↑; alpha-Ketoglutaric acid↑ | 0.611 |
| 40 | Arginine biosynthesis | alpha-Ketoglutaric acid↑ | 1.000 |
| 41 | Glycine, serine and threonine metabolism | Betaine↑ | 1.000 |
| 42 | Glyoxylate and dicarboxylate metabolism | alpha-Ketoglutaric acid↑ | 1.000 |
| 43 | Butanoate metabolism | alpha-Ketoglutaric acid↑ | 1.000 |
| 44 | Prolactin signaling pathway | Estrone↓ | 1.000 |
| 45 | Mineral absorption | Methionine↓ | 1.000 |
| 46 | Central carbon metabolism in cancer | Methionine↓ | 1.000 |

**Table S10. Enriched pathways and significantly different metabolites in negative mode in the comparison of SW vs. GF at 90 days after parturition**

| No. | Metabolic pathways | Significantly different metabolites | P-value |
| --- | --- | --- | --- |
| 1 | Vitamin digestion and absorption | Menaquinone↑; Riboflavin-5-phosphate↑ | 0.072 |
| 2 | Galactose metabolism | Inositol↑ | 0.274 |
| 3 | Ascorbate and aldarate metabolism | Inositol↑ | 0.274 |
| 4 | Arginine biosynthesis | Citrulline↑ | 0.274 |
| 5 | Caffeine metabolism | Theophylline↑ | 0.274 |
| 6 | Cysteine and methionine metabolism | S-Sulfo-L-cysteine↑ | 0.274 |
| 7 | Inositol phosphate metabolism | Inositol↑ | 0.274 |
| 8 | Riboflavin metabolism | Riboflavin-5-phosphate↑ | 0.274 |
| 9 | Drug metabolism - cytochrome P450 | 2-Propylglutaric acid↓ | 0.274 |
| 10 | Phosphatidylinositol signaling system | Inositol↑ | 0.274 |
| 11 | Longevity regulating pathway | Resveratrol↓ | 0.274 |
| 12 | Arachidonic acid metabolism | Prostaglandin G2↓; Lipoxin B4↓; 12(S)-HETE↓ | 0.381 |
| 13 | Biosynthesis of amino acids | Tryptophan↑; S-Sulfo-L-cysteine↑; Citrulline↑ | 0.381 |
| 14 | Oxidative phosphorylation | Riboflavin-5-phosphate↑ | 0.476 |
| 15 | Glycine, serine and threonine metabolism | Tryptophan↑ | 0.476 |
| 16 | ABC transporters | Inositol↑ | 0.476 |
| 17 | Ovarian steroidogenesis | 12(S)-HETE↓ | 0.476 |
| 18 | Aldosterone synthesis and secretion | 12(S)-HETE↓ | 0.476 |
| 19 | Mineral absorption | Tryptophan↑ | 0.476 |
| 20 | Tryptophan metabolism | Xanthurenic Acid↑; Tryptophan↑ | 0.609 |
| 21 | Phenylalanine, tyrosine and tryptophan biosynthesis | 3-Hydroxybenzoic acid↓; Tryptophan↑ | 0.609 |
| 22 | Ubiquinone and other terpenoid-quinone biosynthesis | Menaquinone↑ | 1.000 |
| 23 | Aminoacyl-tRNA biosynthesis | Tryptophan↑ | 1.000 |
| 24 | Platelet activation | Prostaglandin G2↓ | 1.000 |
| 25 | Serotonergic synapse | Prostaglandin G2↓; Tryptophan↑ | 1.000 |
| 26 | Protein digestion and absorption | Tryptophan↑ | 1.000 |
| 27 | African trypanosomiasis | Tryptophan↑ | 1.000 |

**Table S11. Enriched pathways and significantly different metabolites in positive mode in the comparison of SW vs. SF at 90 days after parturition**

| No. | Metabolic pathways | Significantly different metabolites | P-value |
| --- | --- | --- | --- |
| 1 | Prostate cancer | Androstenedione↓; Dehydroepiandrosterone↓ | 0.068 |
| 2 | Endocrine resistance | Androstenedione↓ | 0.093 |
| 3 | Ferroptosis | L-Cystine↑ | 0.093 |
| 4 | Pyrimidine metabolism | 5-Methylcytosine↓; Thymine↑ | 0.097 |
| 5 | Ovarian steroidogenesis | Androstenedione↓; Dehydroepiandrosterone↓ | 0.097 |
| 6 | Pathways in cancer | Androstenedione↓; Dehydroepiandrosterone↓ | 0.097 |
| 7 | ABC transporters | L-Cystine↑; Betaine↑ | 0.128 |
| 8 | Cysteine and methionine metabolism | L-Cystine↑ | 0.179 |
| 9 | Glycine, serine and threonine metabolism | Betaine↑ | 0.257 |
| 10 | Lysine degradation | Pipecolic acid↓ | 0.257 |
| 11 | Prolactin signaling pathway | Androstenedione↓ | 0.257 |
| 12 | Arginine and proline metabolism | Creatinine↓ | 0.329 |
| 13 | Tryptophan metabolism | Kynurenic acid↓ | 0.453 |
| 14 | Protein digestion and absorption | L-Cystine↑ | 0.507 |
| 15 | Steroid hormone biosynthesis | Androstenedione↓; Dehydroepiandrosterone↓ | 0.643 |
| 16 | Metabolic pathways | Kynurenic acid↓; Creatinine↓; Androstenedione↓; Betaine↑; Pipecolic acid↓; Thymine↑; 4-Hydroxybenzaldehyde↑; Dehydroepiandrosterone↓ | 0.720 |

**Table S12. Enriched pathways and significantly different metabolites in negative mode in the comparison of SW vs. SF at 90 days after parturition**

| No. | Metabolic pathways | Significantly different metabolites | P-value |
| --- | --- | --- | --- |
| 1 | Pyrimidine metabolism＊ | Thymidine↑; 2-Deoxyuridine↑ | 0.043 |
| 2 | Nicotinate and nicotinamide metabolism | beta-Nicotinamide mononucleotide↓ | 0.267 |
| 3 | Protein digestion and absorption | 4-Methylphenol↓ | 0.267 |
| 4 | Biosynthesis of unsaturated fatty acids | Lignoceric Acid↑ | 1.000 |
| 5 | Metabolic pathways | beta-Nicotinamide mononucleotide↓; Thymidine↑; Terephthalic acid↓; 4-Methylphenol↓ | 1.000 |
